# The Lived Experiences of Autistic Mothers: A Systematic Review and Thematic Synthesis of Qualitative Evidence

**DOI:** 10.1177/23969415251343850

**Published:** 2025-07-07

**Authors:** Deanne Christie Lockington, Fiona Gullon-Scott

**Affiliations:** 5994 Newcastle University, Newcastle Upon Tyne, UK

**Keywords:** Autism, autistic parenting, systematic review, autistic mother

## Abstract

**Background:**

Increasingly, research has explored autistic mothers’ experiences of motherhood. However, understanding is largely based on single studies. Existing syntheses of qualitative and quantitative research are highly specific, focusing on pregnancy, sensory, infant feeding, and perinatal periods. Thus, a review taking a broader perspective which encapsulates autistic mothers’ experiences beyond early motherhood is warranted.

**Aims:**

To systematically identify, appraise, and synthesize existing qualitative research on autistic mothers’ experiences of motherhood to enrich understanding, and guide future research and practice.

**Methods:**

A systematic review following Preferred Reporting Items for Systematic Reviews and Meta-Analyses (PRISMA), and a qualitative synthesis of extant peer-reviewed qualitative studies and grey literature sources using Thomas and Harden Thematic Synthesis. Methodological rigor was assessed using the Critical Appraisal Skills Programme (CASP) checklist.

**Results:**

Three themes representing the collective experiences and perspectives of 629 autistic mothers from 23 primary studies were developed: “The Embodied Autistic Experience of Motherhood,” “Navigating the Non-Autistic World as an ‘Other’ Mother” and “Recalibrating Identities.”

**Conclusions:**

Autistic mothers report having unique autistic strengths and prioritizing their children. However, their experiences of motherhood are largely colored by autism-specific and identity-related challenges, and adverse experiences related to systemic, structural, and societal othering, specifically, from feeling policed, pathologized and overpowered by professionals. This translates into an increased prevalence of psychological difficulties and need for support. Further research, professional training, systemic changes, and societal awareness are urgently needed to inform understanding and support.

## Introduction

### Autism

Autism is a lifelong neurodevelopmental disability (National Autistic Society [NAS], 2024). It manifests as heterogenous differences in social communication and interaction, alongside specialized, focused interests and repetitive behaviors (American Psychiatric Association [APA], 2022; Cherewick & Matergia, 2023). Consequently, autistic people experience the world differently to non-autistic people and have different lived experiences (Lobregt-Van Buuren et al., 2021; NAS, 2024).

### Research Context

While autism manifests differently for each autistic person, there are nuanced similarities and differences between the experiences of specific groups of autistic people based on shared aspects of their autistic profiles, socio-cultural contexts, and other facets of identity including age and sex (Hamdani et al., 2023; Mo et al., 2022). Understanding the lived experiences of groups currently under-represented in research, including autistic women, is a priority for the autism community, service providers and researchers (Pellicano et al., 2014).

Historically, autistic women have been largely neglected in research, with autistic men and children disproportionally overrepresented until recent years (D’Mello et al., 2022; Lai et al., 2015; Milton, 2014), and remain underrepresented within prevalence data, with the male-to-female sex ratio typically reported as 4:1 yet predicted to be 3:4 (Loomes et al., 2017). These discrepancies are predominantly ascribed to the gendering of autism (Kelly et al., 2022; Watkins, 2014). For example, autism is stereotypically assumed a male or childhood diagnosis and thus not typically associated with females or adult women (Milner et al., 2019). There are indicated sex differences in phenotypic presentations, whereby the female experience of autism reportedly differs from males (Kirkovski et al., 2013), although it is unknown whether this is inherent to autism or a result of socio-cultural context and wider female experiences and expectations. Whatever the explanation, gender-biased diagnostic or screening tools can mean autistic women who meet clinical threshold are at disparate risk from misdiagnosis, delayed diagnosis, or non-diagnosis (Happe & Frith, 2020) because defining criteria are based on males. These factors perpetuate the under-representation of autistic women in research, practice, and society (D’Mello et al., 2022; Taylor & DaWalt, 2020).

To bridge this gap, increase understanding, representation, and clinical relevance to autistic women, further research is needed on their gender-specific experiences (Milner et al., 2019; Pellicano et al., 2014; Taylor & DaWalt, 2020). This may also inform support and reduce diagnostic challenges (Cook et al., 2024; Gosling et al., 2023; Milner et al., 2019).

### Autistic Women

Research has begun to address this gap in the literature by exploring autistic women's experiences in employment, education, and relationships (Baldwin & Costley, 2016; Hurlbutt & Chalmers, 2004; Kanfiszer et al., 2017; Kock et al., 2019), of diagnosis, identity, and support (Harmens et al., 2022; Leedham et al., 2020), masking (Bargiela et al., 2016; Hull et al., 2020) and hormonal changes (Moseley et al., 2020; Steward et al., 2018).

Findings show autistic women's lives are marked by difficulties, disadvantages, abuse, and exploitation across various contexts, and they endure a long-term, complex journey of understanding and integrating their autistic identities (Cazalis et al., 2022; Gosling et al., 2023; Harmens et al., 2022; Kelly et al., 2022). They also face barriers to support, including poor understanding of autism amongst professionals and inaccessible services, meaning their needs are often unrecognized and unmet (D’Mello et al., 2022; Wassell & Burke, 2022).

Overall, research suggests autistic women have gender-unique experiences and are vulnerable to adverse experiences due to their intersectional identities: being autistic and female (Gerschick, 2000; Saxe, 2017). This stems from societal stigma, discrimination, stereotypical expectations, and systemic unawareness (Gosling et al., 2023; Kelly et al., 2022), and is linked with an increased prevalence of psychological challenges (Paricos et al., 2024; Yau et al., 2023).

### Autistic Mothers

While understanding is broadly increasing about autistic women's experiences, specific subgroups of this population, including autistic mothers, remain under-researched (Hernandez-Gonzalez et al., 2023; Pohl et al., 2020). This may be exacerbated by normative parenting discourses that assume disabled people, including autistic mothers, lack capacity to parent or provide adequate childcare (Olsen & Wates, 2003; Wates, 2003).

Emerging research indicates that autistic mothers have mixed experiences of motherhood (Dugdale et al., 2021; Pohl et al., 2020). Some autistic mothers report positive experiences, describing motherhood as joyful and rewarding (Donovan et al., 2023; Dugdale et al., 2021; Hampton et al., 2022a; 2022b). They relish the intense connections with their children, and their ability to care for, and support them (Dugdale et al., 2021). Autistic mothers report higher parental efficacy than non-autistic mothers (Lau et al., 2016) and attribute this, and their parenting strengths, including understanding, relating to, and prioritizing their children, and implementing structure and routines, to being autistic (McDonnell & DeLucia, 2021; Pohl et al., 2020; Winnard et al., 2022).

Conversely, autism presents multiple challenges for autistic mothers which contribute to negative experiences of motherhood (Dugdale et al., 2021; Pohl et al., 2020). These experiences are considered specific to being autistic, and therefore differ from challenges experienced by non-autistic mothers (Donovan et al., 2023; Talcer et al., 2023). For example, autistic mothers report heightened sensory and emotional experiences (Grant et al., 2022; McDonnell & DeLucia, 2021), and communication and executive functioning difficulties (Ferrara et al., 2023; Murphy, 2021; Samuel et al., 2022), which contribute to increased maternal stress and anxiety (Hampton et al., 2022a, 2022b, 2022c; Pohl et al., 2020).

Moreover, research suggests autistic mothers’ challenges are exacerbated by negative interactions with professionals and systems, which is less frequently reported by non-autistic mothers (McDonnell & DeLucia, 2021; Stuart & Kitson-Reynolds, 2024). Autistic mothers are more likely to experience harmful judgements or discrimination regarding their parenting abilities, inaccessible support, and poor understanding about maternal autism (Benson, 2023; Hampton et al., 2022c; Radev et al., 2023; Sanchez, 2023). This can lead to unnecessary parenting assessments, safeguarding procedures, and parental blame (Clements & Aiello, 2021; Dugdale et al., 2021; Griffiths et al., 2019; Murphy, 2021), precipitating and exacerbating psychological challenges including anxiety and depression (Ferrara et al., 2023; Hampton et al., 2022c; Pohl et al., 2020).

### Rationale for Current Review

Overall, research indicates that autistic mothers’ experiences of motherhood differ to non-autistic mothers by virtue of being autistic (Talcer et al., 2023), and their challenges predominantly relate to being autistic. This includes both innate autistic differences and societal challenges (Fukui et al., 2023; Pohl et al., 2020). However, current understanding is based on a limited number of qualitative, quantitative, and mixed-methods studies. Quantitative measures are increasingly contested in autism research, often designed for autistic people not with them (Jones, 2022). This can increase risk of researcher bias and misinterpretation, sometimes resulting in inaccurate conclusions, reducing the clinical relevance of autism research (Botha & Cage, 2022; Jones, 2022).

Qualitative methods, however, are recommended for and by the autism community, particularly when exploring under-researched topics with under-represented groups (Pellicano et al., 2014, 2022). They directly capture autistic people's voices, their nuanced experiences, and leverage their strengths, leading to individualized understanding (Grant & Kara, 2021). These methods are frequently complimented by participatory approaches which involve autistic people in co-developing research, leading to more meaningful outcomes (Benevides et al., 2020). Furthermore, qualitative quality appraisal tools enable thorough assessment of researcher reflexivity, reducing bias and increasing trustworthiness (Johnson et al., 2020).

To ensure autistic mothers’ voices are better represented, qualitative, participatory research is needed, including primary studies and reviews of existing evidence (Gotham et al., 2015; Roche et al., 2020). Additional primary research is recommended, however, amalgamating existing evidence can enrich understanding by illustrating what is known and remains undiscovered (Noyes et al., 2021). Although several reviews exist, most are highly specific, focusing on topics including infant feeding (Grant et al., 2022), pregnancy and social-medical considerations (Ferrara et al., 2023), sensory challenges during pregnancy and childbirth (Samuel et al., 2022) and early motherhood (McDonnell & DeLucia, 2021; Stuart & Kitson-Reynolds, 2024). Other, broader syntheses focus on autistic mothers and fathers experiences of parenthood, from conception to relationships with adult children, (Thom-Jones et al., 2024), or exclude recent research (Turner, 2021). Therefore, an updated, broader review solely exploring autistic mothers’ overall experiences is warranted.

This review aimed to identify all available qualitative evidence on autistic mothers’ self-reported experiences of motherhood and unify their diverse first-hand accounts through a qualitative synthesis, providing a novel picture of extant literature. Synthesizing qualitative research presents an interesting juxtaposition; it is time, context, and population specific and attempting to generalize it risks de-contextualizing findings. However, drawing it together provides deeper understanding which has valuable implications for policy, practice, and research (Newman et al., 2006). Thus, qualitative synthesis methods amalgamate primary qualitative research findings and offer novel interpretations based on the findings as a “whole” (Thorne et al., 2004, p. 1385) to produce a result “greater than the sum of its parts” (Barnett-Page & Thomas, 2009, p. 2).

### Aims

This review aims to synthesize evidence on autistic mothers’ experiences of motherhood by identifying, describing, critically appraising, and thematically synthesizing existing qualitative research that has explored their first-hand accounts. It hopes to enhance understanding, provide new or more meaningful insights, address important knowledge gaps and reveal what remains unexplored, to guide research and practice (Grypdonck, 2006; Noyes et al., 2023). The review question was formulated using the Population, Interest, Context (PICo) framework (Appendix A): “what is known about autistic mothers’ lived experiences of motherhood?” (Noyes et al., 2023).

This review was situated within the neurodiversity paradigm and approached from a Critical Realist (CR) epistemological perspective. CR focuses on understanding, rather than solely describing social reality (Vincent & O’Mahoney, 2018). It assumes that knowledge of reality is mediated by our beliefs and perceptions and emphasizes the importance of context (Maxwell & Mittapalli, 2010; Oliver, 2012).

## Methods

### Scoping, Protocol, and Registration

This review was undertaken following Preferred Reporting Items for Systematic Reviews and Meta-Analyses (PRISMA) checklist (Page et al., 2021) and The Enhancing Transparency in Reporting the synthesis of Qualitative Research (ENTREQ) guidelines (Moher et al., 2009; Tong et al., 2012). The PRISMA checklist was chosen as it facilitates transparent and complete reporting of systematic reviews, allowing decision makers to assess the trustworthiness and applicability of review findings (Page et al., 2021). It is well established, and widely endorsed for different types of reviews exploring a broad range of topics (Page & Moher, 2017; Page et al., 2021). However, additional guidelines should be consulted when using PRISMA for qualitative synthesis, as the processes often differ from quantitative and mixed-methods syntheses PRISMA was primarily designed for (Page et al., 2021). Therefore, the ENTREQ guidelines were followed to further increase the conduct, reporting and transparency of this review, as recommended (Moher et al., 2009; Tong et al., 2012).

Originality was established by searching Prospero, Cochrane, and Google Scholar. Existing reviews on the topic were identified (Ferrara et al., 2023; Grant et al., 2022; McDonnell & DeLucia, 2021; Samuel et al., 2022; Stuart & Kitson-Reynolds, 2024; Thom-Jones et al., 2024; Turner, 2021) although none had the same aims. A review protocol was developed and registered on PROSPERO (reference: CRD42024496375) to increase transparency and reduce bias (Pieper & Rombey, 2022).

### Eligibility Criteria

This review aimed to thematically synthesize qualitative literature to capture autistic mothers’ first-hand accounts. Studies were included if they: (a) used qualitative data collection and analysis methods, (b) reported data exclusively from first-hand accounts of self-identifying or clinically diagnosed autistic mothers in context of their experiences of any period of motherhood (including pregnancy) and/or parenting at least one child (c) adhered to the central tenets of scientific rigor (d) were available in English, and (e) were published after 1911, the year autism was coined (Kanner, 1943).

Qualitative studies including both autistic and non-autistic mothers, or both autistic mothers and fathers were included if the data collection, analysis, and findings were clearly demarcated between groups. Mixed-methods studies were included if qualitative data collection and analysis were clearly demarcated from quantitative data and analysis. Only qualitative data from autistic mothers pertaining to their parenting experiences were extracted and analyzed.

Qualitative studies within the “grey literature” were included if they: (a) adhered to central tenets of scientific rigor and (b) were approved by educational or professional research committees (e.g., unpublished MSc, Post-Graduate, Doctoral or PhD studies). These studies were considered to expand the body of available literature which could contribute towards the synthesis, particularly given limited research in the area. The primary reviewer manually screened studies using the eligibility criteria. Further detail is provided in Appendix B.

### Information Sources

A range of information sources were included to increase the likelihood of capturing all available literature (Thomas & Harden, 2008; Tong et al., 2012). Seven electronic bibliographical databases were searched in December 2023: (a) APA PsycINFO via Ovid (1806–2023), (b) CINAHL, (c) EMBASE via Ovid (1974–2023), (d) Scopus, (e) MEDLINE via Ovid (1946–2023), (f) PubMed and (g) British Education Index via EBSCOhost, reflecting the array of academic disciplines contributing to research on the topic. Grey literature searches were completed in January 2024 using (a) ProQuest and (b) The International Society for Autism Research (INSAR) congress archives. Citations of relevant studies were hand-searched to expand the search.

### Search Strategy

Search terms were developed by assimilating keywords on the topics of motherhood and autism from relevant articles. Keywords were refined through scoping searches within selected databases. Final search terms consisted of one category describing motherhood and one describing autism. Categories were defined using a combination of search terms (Appendix C), input as keywords and mapped to suggested subject headings within databases. Categories were combined using the Boolean operator “AND.” The combination of search terms within the categories were combined with Boolean operator “OR.” The same search terms and limiters were used and adapted accordingly across databases, and for grey literature searches.

### Selection Process

Searches were first completed in bibliographical databases. Identified records were collated and exported to Endnote 20 and duplicates removed. Records were individually screened for relevance by title then abstract against the eligibility criteria by each reviewer. Where records appeared to meet criteria, full text was obtained and reviewed. For instances where it was unclear whether studies met criteria, discussion took place between authors and decisions made accordingly.

Grey literature searches were completed afterward. Material was screened for relevance by title, abstract or summary, where provided, on initial results pages against the same eligibility criteria. A PRISMA (Page et al., 2021) flow chart was used to guide this process.

### Data Collection

A data collection form (Appendix D) and table was designed to collect and present the extracted data of interest based on the reviews’ question and objectives. The primary reviewer completed this process manually. Data included: study information (e.g., title, authors, publication year, country, type) focus (e.g., parenting period, nature of experiences), sample (e.g., population, sample size, sampling method, recruitment, eligibility criteria, participant characteristics), methods (e.g., design, epistemology, researcher reflexivity, data collection and analysis) and “key findings” (e.g., themes, summary).

Given the diverse reporting and presentation of qualitative research, the “key findings” section of the data extraction table initially comprised all data identified as “results,” “themes,” or “findings” in the abstract or main body of primary papers which related to the review question (Noyes et al., 2023; Sandelowski & Barroso, 2002). It was later transferred to Excel for purpose of synthesis and summarized within the extraction table. “Summary” data comprised of a summary of primary authors conclusions from the primary reviewer's perspective. This was checked and verified by the second author.

All data collected was considered pertinent to answer the review question and aid quality appraisal. Researcher reflexivity data were important in context of this review's aim to capture autistic mothers’ voices, the thematic synthesis method employed and the CR epistemology guiding this review. Thomas and Harden (2008) suggest the extent to which researchers reflect on their potential influences and prioritize participants’ voices aids appraisal of the quality and trustworthiness of findings and supports the analytical process. Within this, they acknowledge the meaningful contribution of researchers, and the reviewers’ subjective interpretations. Similarly, CR views knowledge as subjectively, relatively, and contextually constructed by individuals, acknowledging that a reality exists independent of our knowledge of it, but that all constructions of reality are equally valid (Tikly, 2015).

### Critical Appraisal

The Critical Appraisal Skills Programme (CASP) checklist for qualitative studies (CASP, 2018) was used to assess studies’ methodological rigor (Appendix E) (Noyes et al., 2021). This compromises 10 domains to assess the quality of qualitative studies, and qualitative components within mixed-methods studies irrespective of method, design, and epistemological position. This included peer-reviewed studies and grey literature sources given they all adhered to central tenets of scientific rigor and were approved by educational or professional research committees.

Studies were assessed and graded individually against the checklist by the primary reviewer, and half were reviewed by the second reviewer. A total score was calculated for each paper to illustrate overall quality, color-coding was used to depict areas of strengths, uncertainty, and weaknesses. All papers were included irrespective of quality rating, as no standardized criteria or guidelines exist to classify or rank studies quality or exclude them on this basis (Noyes et al., 2019).

### Synthesis Methodology

Thematic synthesis offers a distinct three-stage process that distinguishes between reviewing and synthesizing primary data from diverse sources with varying epistemologies and “going beyond” to interpret it. It preserves the uniqueness, richness, and context of primary studies, while acknowledging reviewers’ meaningful contribution to generating new hypotheses through connecting patterns of shared meaning across datasets to answer questions about people's diverse subjective experiences (Thomas & Harden, 2008). Although many qualitative methods exist, thematic synthesis was deemed the most suitable in context of the Review question, Epistemology, Time/Timescale, Resources, Expertise, Audience and purpose, Type of data (RETREAT) framework (Booth et al., 2016; Tong et al., 2012).

**Figure 1.**
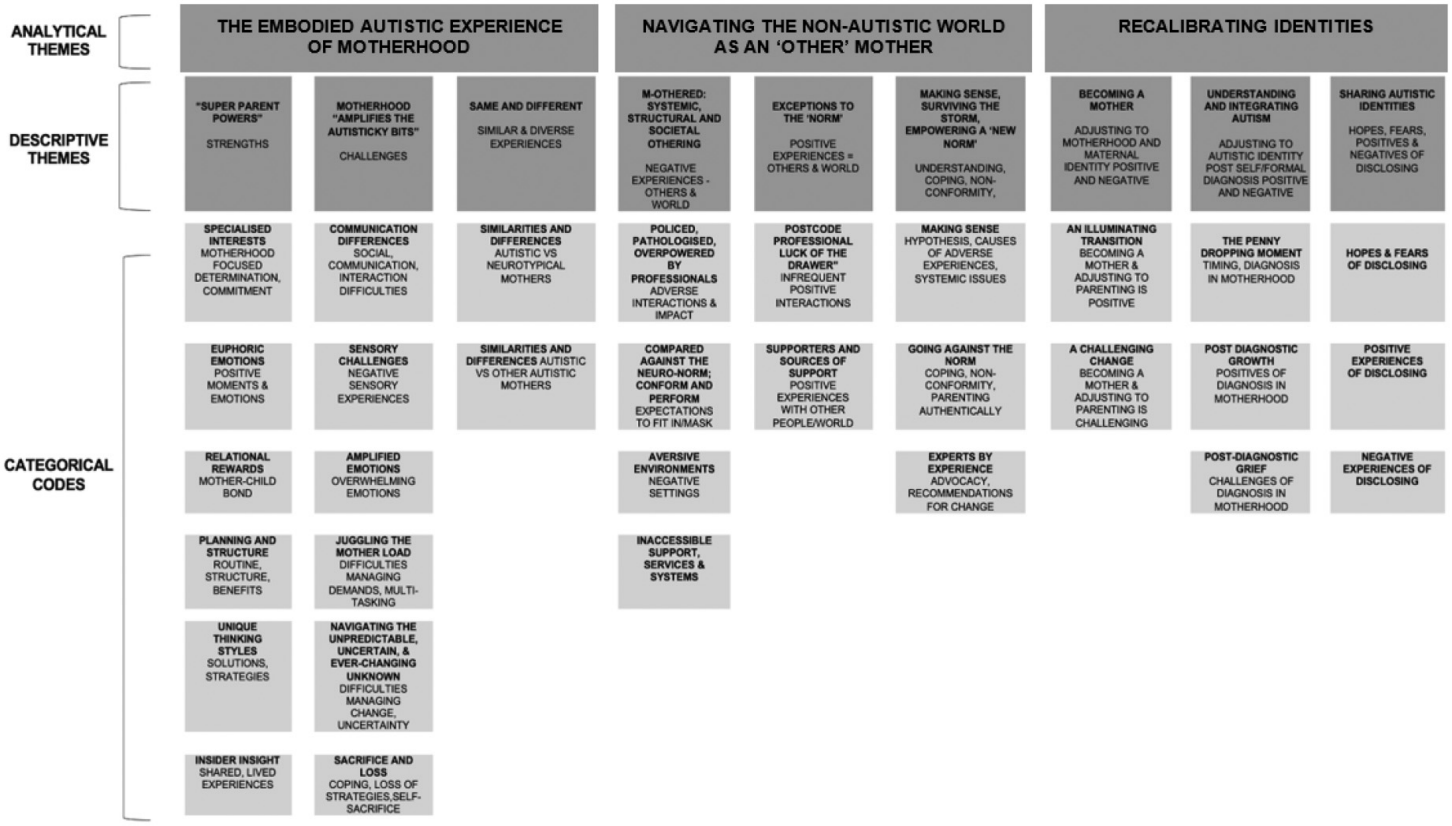
Thematic Framework.

Thomas and Harden's (2008) three-stage process was followed to review and synthesize findings from all primary studies. Given all grey literature sources had a degree of scientific rigor and could be critically appraised, findings were synthesized with published literature. In stage one, the “key findings” were transferred into an excel document by the primary reviewer, this data was checked and verified by the second reviewer. Each line of text was allocated to an individual row within one column, and initial codes developed inductively according to the meaning and content. Some lines of text were initially allocated multiple codes where concepts overlapped (Thomas & Harden, 2008). This resulted in 133 codes then organized by shared patterns of meaning and condensed into 31 codes. In stage two, codes were mapped onto nine descriptive themes. In stage three, themes were grouped according to similarities and differences, and three analytical themes generated to reflect their content (Figure 1). The process of coding and theme development was an iterative process, completed collaboratively by both reviewers.

### Reflexive Statement

Systematic reviews aim to minimize bias and maximize transparency by following and reporting on a standardized series of steps, as documented above (Menon et al., 2022). However, reviewers also need to acknowledge the personal, interpersonal, epistemological, methodological, and contextual factors influencing their work to enhance trustworthiness (Dodgson, 2019; Olmos-Vega et al., 2022; Tod et al., 2022). This aligns with the CR epistemology underpinning this review, and the thematic synthesis approach employed (Maxwell & Mittapalli, 2010; Oliver, 2012; Thomas & Harden, 2008).

The primary reviewer was a Trainee Clinical Psychologist with previous clinical experience, a passion to increase awareness and understanding, and advocate for the needs of under-represented, neurodivergent groups to inform support and systemic changes. They are a neurodivergent woman and non-mother, with personal and professional relationships and experiences working therapeutically with neurodivergent people. This influenced their understanding of the strengths and challenges of neurodivergent populations, and the nature and impact of their lived experiences and socio-cultural contexts. It also influenced their perspectives on autism, including their neurodiversity affirming stance. This recognizes neurodivergence, including autism, as a natural variation in human diversity, embraces neurodivergent people's unique strengths and differences and advocates for them to have equal rights, voice, and agency in their own lives (British Psychological Society [BPS], 2024).

The reviewer's stance influenced many aspects of this review. For example, they used identity-first and neuro-affirming language preferred by the autistic community. They reflected on the impact of selecting non-neurodiversity affirming illustrative quotes to support the themes including how they could reflect participants’ realities yet inadvertently reinforce harmful narratives and discriminatory ideologies, potentially negatively impacting them. Including participants’ reported strengths and self-affirmative statements relating to their parenting and autism helped to counter any potential bias and present a more balanced, neutral perspective. Incorporating participants’ views on factors contributing to their experiences, and specific suggestions for coping or change, was also important to inform the reviews recommendations, and the researchers’ interests in increasing awareness and advocating for change. Moreover, it helped to ensure conclusions and recommendations were meaningful, reflective of participants’ voices and co-produced, aligning with autism research recommendations (Kaplan-Kahn & Caplan, 2023).

The reviewer also reflected on the influence of their stance in relation to the theoretical context, epistemology, and timing of this review. This review was conducted during a period of evolving perspectives on autism, transitioning away from dominant, traditional medical paradigms focused on “curing” autism “disorder” and toward an affirming understanding of autism as an identity, difference, and disability within the neurodiversity paradigm (Pellicano et al., 2022; Singer, 1999). The review topic presented a novel opportunity for the reviewer to explore the experiences of a group who may be caught in the crossfire of these evolving perspectives from their neurodiversity affirming stance within context of the neurodiversity paradigm, with a view to inform recommendations for practice and future research.

The CR epistemology meant that participants perspectives were presented as a valid construction of their realities, irrespective of whether they were accepted by others (Oliver, 2012). This was important given the reported under-representation of this group and the dominant discourses around autism and motherhood. The review was completed as research on the topic was increasing, and reports of increased discrimination toward autistic mothers and its impact were emerging. This indicated the review was timely, of importance and clinical relevance to autistic mothers and those working with this group. However, it potentially increased the risk for new papers to be missed, highlighting a potential limitation of this review.

This review was undertaken as part of a wider project which included an empirical study exploring the lived experiences of autistic mothers whose parenting was questioned and/or challenged by professionals (Lockington & Gullon-Scott, 2025). Phase one involved a PPI group with six autistic mothers with lived experience of the topic, phase two involved semi-structured interviews with thirteen autistic mothers with lived experience of the topic. PPI group participants informed the development of the project, expressed a need for more research on the area, and an updated understanding of what research exists and tells us. This, alongside previous research recommendations, informed the rationale for the current review and ensured autistic mothers were involved in shaping it.

## Results

Throughout this section, studies are referenced by number as illustrated in Table 2.

### Study Selection

**Figure 2.**
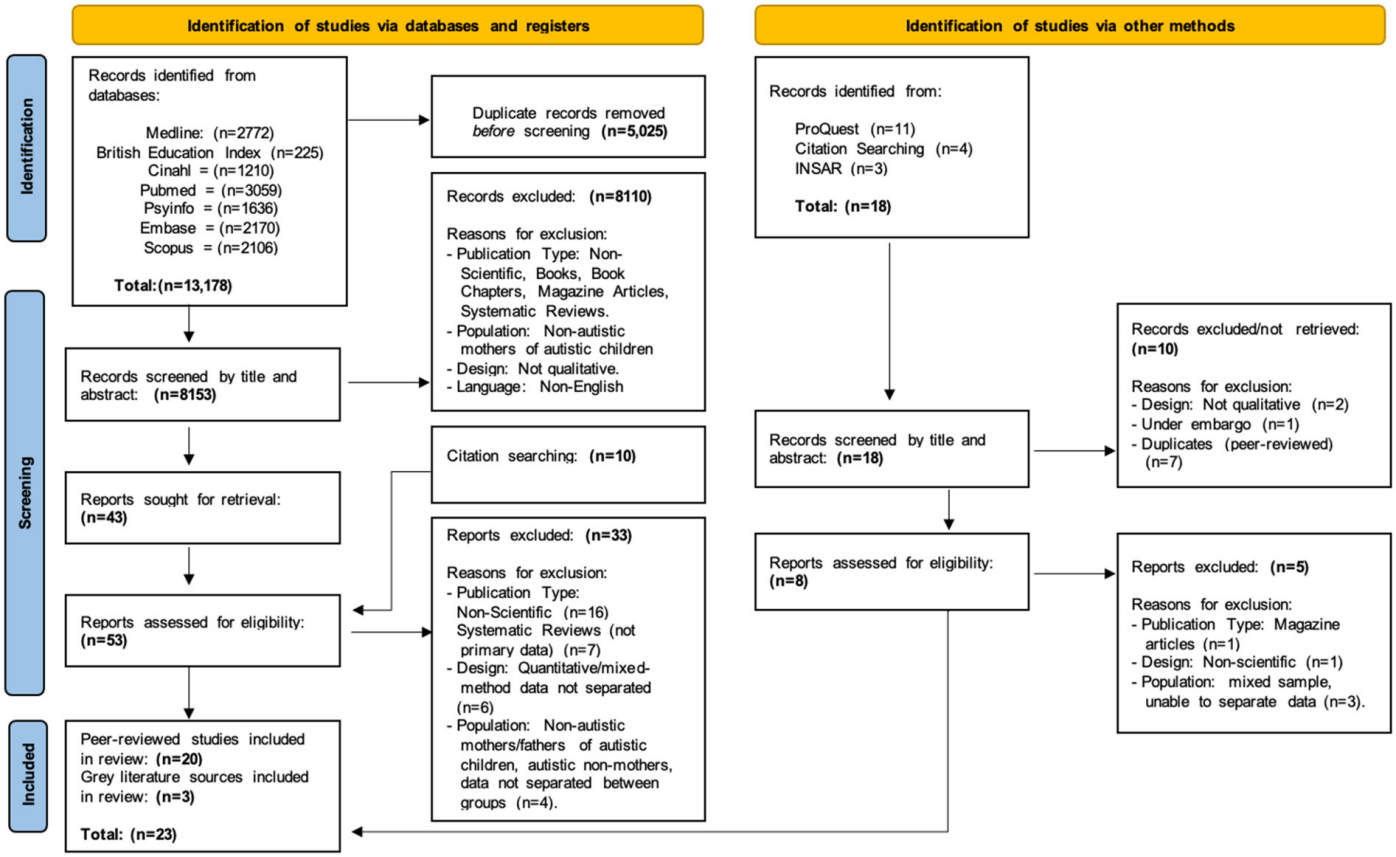
PRISMA 2020 Flow Diagram for New Systematic Reviews, which Included Searches of Databases, Registers, and Other Sources.

The screening and selection process are illustrated in a PRISMA diagram (Figure 2) and summarized below (Page et al., 2021).

**Figure 3.**
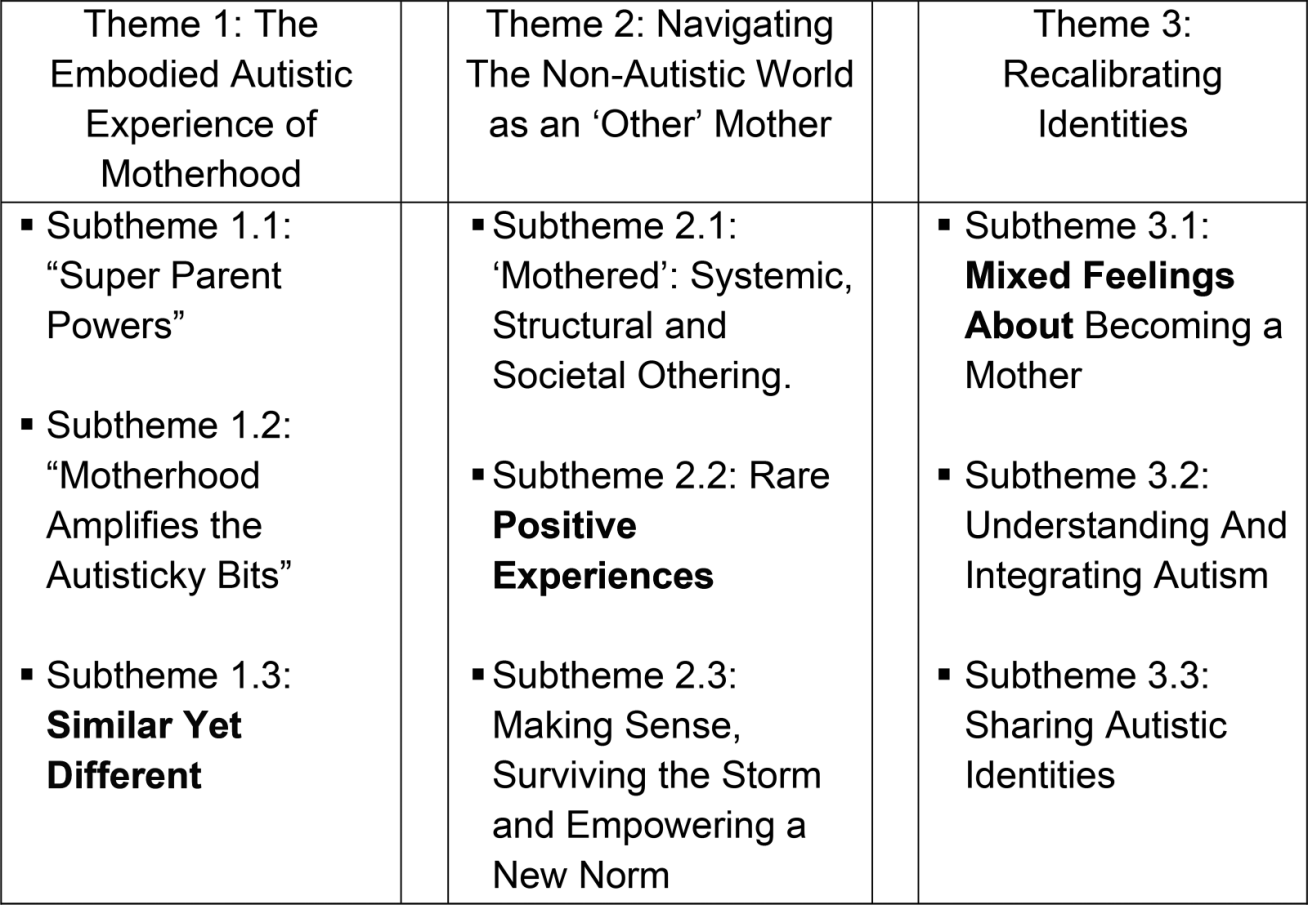
Visual Depiction of Analytical Themes and Descriptive Subthemes.

Database searches identified 13,178 records; 5025 duplicates were removed. A total of 8153 records were screened by title and abstract, 8110 of which were excluded. The full text of the remaining 43 records were reviewed against the eligibility criteria, along with 10 records identified via citation searching. Some required further discussion with the review team (Appendix F). Following this, 33 studies were excluded. The remaining 20 were deemed eligible and included in this review, including 16 studies from database searching and 4 from citation searching.

Grey literature searches identified 18 records, all of which were reviewed by title and abstract or summary where provided, 10 were excluded. The remaining eight reports were screened in full. A further five were excluded. A total of three reports were deemed eligible for inclusion. Overall, a total of 23 peer-reviewed papers and grey literature sources met eligibility and were included.

### Study and Sample Characteristics

An overview of study and sample characteristics are presented alphabetically by Author (Table 1) and discussed in context of their respective data source thereafter. Detailed information is provided in Appendix G.

**Table 1.** Summary of Studies Included in the Synthesis.

No.	Study Information		Sample	Methods		Findings	
	Author Year Country Type	Parenting Period Nature of Experience	Population Sample Size	Design Epistomology Reflexivity Participation	Data Collection and Analysis	Main Themes	Summary
1	Benson (2023) UK. Peer-Reviewed Article	No specific time period. Social work services and interventions.	Autistic mothers (*n* = 7).	Qualitative design. Critical constructivist. Positionality statement. Meaningful participatory approach	Semi-structured interviews Grounded Theory	1. Perplexing children and cognitive injustice 2. Neurodivergent children and the battleground of school 3. Support? Here is a parenting course. Not bloody boundaries again 4. Epistemic authority and the “normal”	Participants and their children are viewed as “perplexing” through a neuro-normative lens of social work scrutiny. Challenges and adverse experiences were located within participants rather than the wider systems, structures and processes which make autism disabling.
2	Burton (2016). UK, Unpublished Doctoral Thesis (Grey Literature)	No specific time period. Pregnancy, birth, and general parenting experiences from birth-adolescence.	Autistic Mothers (*n* = 7).	Qualitative design. Critical Realist (CR). Reflects on CR and feminist stance and influence of power. Meaningful participatory approach.	Semi-structured interviews. IPA	1. We are different 2. Negotiating difference 3. The role of the mother–child relationship. 4. Navigating the parenting journey	Findings highlighted the connection autistic mothers have with their children and sensory and communication challenges. These challenges impacted their experiences throughout their parenting journey, and they perceived themselves differently to others.
3	Donovan (2020) USA. Peer-Reviewed Article	Pregnancy-Perinatal period (2–3 months). Pregnancy, childbirth and delivery and care in acute services and settings.	Autistic mothers (*n* = 24).	Qualitative Interpretative Descriptive Design. Role of researcher and epistemological position unknown. Some stakeholder involvement.	Semi-structured interviews Qualitative analysis method.	1. Having difficulty communicating 2. Feeling stressed in an uncertain environment 3. Being an autistic mother.	Participants childbirth experiences are more stressful than non-autistic mothers due to communication and sensory differences and pre-existing anxiety. Miscommunication with, and negative judgements from nurses had negative implications, resulting in increased anxiety, preventing further attempts to communicate.
4	Donovan et al. (2023). USA Peer-Reviewed Article	Pregnancy-Perinatal period (2–3 months). Post-partum care and experiences in perinatal period.	Autistic mothers (*n* = 24).	Qualitative Interpretative Descriptive Design. Epistemological position unknown, no reflexive statement. Only professional stakeholder involvement.	Semi-structured Interviews. Qualitative analysis method	1. Having difficulty communicating 2. Feeling stressed in an uncertain environment 3. Being an autistic mother	Participants bonding experiences are similar to non-autistic mothers, they expressed love and concern for babies. Some needed time to recover before caring for babies. Childbirth stress and caring demands were exhausting and overwhelming. Concerns within post-partum period and post-partum care included sensory differences, communication difficulties, trouble trusting nurses and negative judgements.
5	Dugdale et al. (2021), UK Peer-Reviewed Article	Childhood and Adolescent Period. General Experiences of Motherhood.	Autistic mothers of children aged 5–15 years (*n* = 9).	Qualitative Design. No reflexive statement researcher locates self in research context Meaningful participatory approach.	Semi-structured interviews IPA	1. Autism fundamentally impacts parenting 2. Battle for the right support 3. Development and acceptance 4. The ups and downs of parenting	Autistic mothers find motherhood a joyous experience and have intense connections and closeness with children. Self-acceptance and self-care are important for personal growth. Autistic mothers’ challenges relate to autism and are unlikely to be experienced by non-autistic mothers. Challenges include negotiating misunderstandings with others, managing demands, negative interactions with professionals and lack of professional awareness, acceptance and support which had a profound negative impact.
6	Gardner et al. (2016). USA, Peer-Reviewed Article	Pregnancy, childbirth, and early post-partum period. Experiences related to early motherhood.	Autistic mothers (*n* = 8).	Qualitative Design. Role of researcher and epistemological position unknown.	Qualitative Questionnaire. Open and holistic coding and analysis methods.	1. Processing sensations 2. Needing to have control 3. Walking in the dark 4. Motherhood on my own terms	Autistic mothers experience several challenges throughout pregnancy, birth and early motherhood including sensory challenges, lack of control, poor communication, difficulties adapting to motherhood and negative experiences with professionals. Professional interactions shaped positive or negative experiences.
7	Gore et al. (2024). Australia. Peer-Reviewed Article	No specific time period. Experiences of employment and parenting and useful daily support	Autistic working mothers (*n* = 10).	Inductive Qualitative Design Subjective epistemology, relativist ontology. Reflexive statement. Participatory approach.	Semi-structured interviews. Inductive RTA	1. Wellbeing: Work gives me purpose; discusses how employment supports mental wellbeing. 2. Challenges: It's hard being an autistic working mother. 3. The invisible disability: Everyone thinks I look okay.	Participants have similar challenges to non-autistic working mothers including stress related to juggling multiple roles, however, have unique challenges related to gender and autism including poor understanding of maternal autism amongst employers and other professionals and managing care and time, particularly with neurodivergent children Employment supports mental wellbeing. Negative judgments and inaccurate assumptions by professionals prevented participants from seeking support.
8	Grant et al. (2023). UK, Peer-Reviewed Article	Maternity period. Experiences of childbirth, infant feeding, and maternity services and associated health care.	Autistic mothers (*n* = 141).	Mixed method design. Locates self in research context Epistemological position unknown.	Mixed method survey. Inductive TA	1. Breastfeeding 2. Formula feeding 3. Infant feeding support.	Autistic mothers have a strong desire to breastfeed. Mixed experiences were reported, challenges were ascribed to bodily changes and sensory processing and interception differences. Receiving breastfeeding support was significantly associated with positive breastfeeding experiences, there were many negative aspects of infant feeding support, and this support did not appear to address Autistic bodily differences that impacted on breastfeeding.
9	Hampton et al. (2022a). UK Peer-Reviewed Article	Third trimester. Pregnancy, physical and sensory experiences, and interactions with healthcare professionals.	Autistic mothers (*n* = 24) and non-autistic (*n* = 21) women.	Qualitative Design. Authors locate self in research context Epistemological position unknown. Some stakeholder involvement.	Semi-structured interviews. Inductive TA	1. Physical and psychological impact of pregnancy 2. The impact of formal and informal support 3. Fears and hopes of motherhood	Autistic mothers experienced heightened sensory and physical symptoms in pregnancy compared to non-autistic mothers. Autistic mothers were sometimes reluctant to disclose their diagnosis to professionals and felt professionals lacked autism knowledge. While both groups appreciated clear information about their care, autistic participants highlighted the need for detailed information, time to process information. and sensory adjustments in healthcare settings.
10	Hampton et al. (2022b). UK, Peer-Reviewed Article	Pregnancy, 2–3 months post-birth. Experiences of childbirth and parenting during post-natal period, post-natal health care and the benefits and challenges of parenthood.	Autistic mothers (*n* = 21) and non-autistic mothers (*n* = 25).	Qualitative Design. Role of researcher and epistemological position unknown. Some stakeholder involvement.	Semi-structured interviews. Inductive TA	1. Positive and negative birth experiences 2. Rewards and challenges of motherhood 3. Impact of formal and informal support	Findings showed sensory aspects of childbirth could be challenging for autistic mothers. They stressed the importance of sensory adjustments and clear, direct communication from professionals during the birth. During childbirth and the post-natal period, autistic mothers sometimes felt professionals lacked knowledge of autism which could hinder receiving appropriate adjustments. Several parenting strengths and challenges were identified.
11	Kwang-Hwang and Heslop (2022) UK Peer-Reviewed Article	No specific time period. Experiences of parenting and parenting support.	Autistic mothers (*n* = 5) and autistic fathers (*n* = 2).	Qualitative Design. Role of researcher and epistemological position unknown. Minimal stakeholder involvement	Focus group. Inductive TA	1. The ups and downs of parenting 2. Misunderstood and negatively judged, 3. Battle for the right support.	Autism may not impact always on mothers parenting capacity, and, when it does, they can succeed in raising their children, especially their autistic children, if they are provided with appropriate support services. Their parenting style and capabilities were misunderstood by professionals who used traditional pathologizing assumptions on parental capacity.
12	Lewis et al. (2021). USA Peer-Reviewed Article	Early motherhood; childbirth Childbirth experiences	Autistic mothers (*n* = 16)	Qualitative Narrative Design. Authors locate self in role, no reflexive statement. Epistemological position unknown. Meaningful participatory approach.	Semi-structured interviews. Narrative Analysis	Tension mostly occurred when healthcare teams acted out of balance with their approach to care. Participants felt their concerns were minimized, their wishes ignored, that they were left out of critical communication and education, and their autistic traits such as sensory sensitivities were out of balance with the birth environment. These impacted their ability to communicate with providers and participate in the birth.	Poor communication, untreated pain, and sensory overload dominated the birth narratives of participants. Autistic mothers’ personal narratives suggested that the way they were treated by health care team members and the social and sensory stimuli in the birthing environment were most influential in shaping their birth stories.
13	Libster (2023) USA Reviewed Article	No specific time period. Experiences of parenting non-autistic adolescent daughter, birth-adolescence.	Autistic mothers (*n* = 7).	Descriptive pilot study. Qualitative design. Participatory approach. Reflective statement. Discussed IPA epistemology. Meaningful participatory approach.	Semi-structured interviews. IPA	1. Closeness in relationships 2. Parenting strengths 3. Identifying own social challenges 4. Building daughters’ social skills.	Autistic mothers have strengths and challenges and guide their children's social development. Strengths include affectionate loving relationships, understanding and supportive mother–daughter relationship and proactive approach daughters’ social development. Challenges include understanding social dynamics, negative experiences with other parents and concerns about daughters’ social development.
14	Marriott et al. (2022). UK Peer-Reviewed Article	No specific time period. General parenting.	Autistic mothers (*n* = 7), autistic fathers (*n* = 1).	Qualitative interpretative descriptive design, Participatory Approach. Reflective statement. Epistemological position unknown.	Semi-structured interviews IPA	1. The interaction of parents’ and children's autistic traits both helps and hinders parent–child relationships 2. The personal impact of being a parent with autistic traits 3. Home is a rare place of acceptance of autistic traits for parents and children 4. Managing the complexities of professional services: struggling to be heard, believed, and supported.	Difficulties include parental mental health and navigating professional services. Novel participant experiences included the interaction between parental and child autistic traits helping and hindering their parenting; parents learning to manage their own autistic traits, and parents finding the home to be an accepting place of autism.
15	Morgan (2019) UK, Unpublished MSc Dissertation (Grey Literature)	Early motherhood; pregnancy- childbirth. Pregnancy and childbirth experiences	Autistic mothers (*n* = 249).	Mixed method design. Contextualist stance within neurodiversity and social model. Minimal self-reflexivity. Meaningful participatory approach.	Online survey. Inductive TA	1. Community and isolation 2. Privacy and freedom 3. Diagnosis—timing and skepticism 4. Retrospect—the effect of timing 5. Exceptional care	The adult autistic population of women who have given birth is evidently underdiagnosed and underserved by current medical care. Autistic women's care experiences were largely negative and included poor-quality practices. Good quality practice examples included positive home transitions, consequences of poor practice included potentially unnecessary interventions and concerns about parenting ability.
16	Pentz et al. (2023). UK, Peer-Reviewed Article	Perinatal period. Experiences of perinatal mental health care services.	Autistic mothers (*n* = 5).	Qualitative Design. Researchers state role, no discussion of influence. Unknown epistemology. Non-participatory approach.	Semi-structured interviews. TA.	1. Interventions, 2. Support of ASC characteristics 3. Practitioners Support,	Highlighted positive aspects of the service and barriers. Participants’ experiences were mixed. Positives include continuous support and flexibility around appointments. Negatives include online format of DBT group and delays in autism diagnostic assessments.
17	Radev et al. (2023). UK. Peer-Reviewed Article	No specific time period. Experiences with statutory services.	Autistic mothers (*n* = 10).	Qualitative interpretative descriptive design Author locates self in research context, fails to discuss influence. Epistemology unknown. Meaningful participatory approach.	Semi-structured interviews IPA	1. The wider system is the problem. 2. Feeling judged and stigmatized	Overall “absolutely awful” experiences with systems including unfair and discriminatory processes and professionals uninformed and outdated views about autism contributed to challenges including being dismissed, unsupported, and poor communication which led to self-reliance. Participants feared disclosing autism or were judged and treated negatively.
18	Rogers et al. (2017). Australia, Peer-Reviewed Article	Pregnancy, birth and early Motherhood Experiences of pregnancy, birth, maternity care, and post-natal period.	Autistic mother (*n* = 1).	Qualitative, case study design. No researcher reflexivity, epistemology unknown.	Semi-structured interviews, TA.	1. Communication and service difficulties 2. Sensory stress 3. Parenting challenges.	Findings suggest autistic women face challenges during pregnancy, birthing, and early mothering. These challenges evolve from perceptions of her from midwives and other caregivers. If a woman perceives that her midwife is judgemental about her, then she may withdraw from the care and support she and her baby need.
19	Sanchez (2023). UK Doctor of Philosophy Thesis (Grey Literature)	No specific time period. Experiences of being an autistic a mother, social expectations, and interactions with professionals and clinicians,	Autistic Mothers (*n* = 12)	Qualitative Design. CR stance. Reflexive statement Meaningful participatory approach.	Semi-structured interviews, RTA.	1. Identity: Knowing I’m autistic helps me to understand myself. 2. Masking: Masking is a real double-edged sword 3. Support, Women like me “fall through the gaps” of support 4. Mothering: A good mum wants the best for her children, 5. Motherhood: autistic mothers are judged and problematized by the same forces that police gender roles in society. 6. Knowledge: -if you are autistic, it's presumed that you don’t know anything about anything	Findings tell a story of the lightbulb moment of participants realizing they were autistic, the challenges of masking and accessing support; the joys and difficulties of mothering, the expectations of motherhood and finding ways to resist and kick back, creative solutions and develop expertize. It highlights that poor awareness, understanding and support of autistic mothers contributes to mother blame narratives despite autistic mothers being highly skilled at recognizing and meeting their children's needs. Findings also show autistic mothers are attempting to resist the neuro-normative narrative of “good mother” and co-construct own ideal of “good autistic mother.”
20	Smit & Hopper, (2023). UK, Peer-Reviewed Article	No specific time period, General Parenting Experiences, Influence of Childhood Trauma, Mental Health, and Wellbeing.	Autistic mothers (*n* = 8), autistic fathers (*n* = 1).	Qualitative Design. Reflexive Statement. Non-participatory approach. Acknowledged double-hermeneutic IPA approach but did not discuss epistemological position.	Semi-structured interviews. IPA	1. Identity and Purpose Love and Joy 2. Looking Through a Lens of Trauma 3. External Factors.	Autistic parents had intimate parent–child connections. Children were sources of love and joy. Parents’ childhood trauma influenced parenting experiences; extreme empathy, perfectionism and drive to protect children from the same trauma. Professionals’ acceptance and awareness of autism was integral for positive outcomes. Mothers experienced pervasive sensory overload from their environments related to losing coping mechanisms when they became parents.
21	Talcer et al. (2023). UK, Peer-Reviewed Article	Early Motherhood Nature and impact of sensory experiences in pre-natal, childbirth and post-birth period.	Autistic mothers (*n* = 7).	Qualitative Design Authors locate self in research context. Epistemological position unknown. Meaningful participatory approach.	Semi-structured interviews. TA.	1. Antenatal experiences 2. Sensory experiences in motherhood 3. The impact of sensory processing difficulties 4. Strategies and needs 5. Diagnosis	Autistic mothers with sensory processing difficulties have extreme and pervasive sensory challenges which exacerbated stress and anxiety making many aspects of motherhood challenging. It impacted multiple facets of their mothering roles, mental and physical health, and ability to plan and organize. Helpful strategies include lessening effects of sensory difficulties, asking for help, and linking with other autistic mothers.
22	Wilson and Andrassy (2022). USA Peer-Reviewed Article	Early motherhood, breastfeeding period. Breastfeeding experiences.	Autistic mothers (*n* = 23).	Qualitative phenomenology design. Researcher role and epistemology unknown.	Semi-structured interviews. TA	1. Intense sensory perception 2. Focused determination 3. One size doesn’t fit all.	Autistic adults can have social interaction and expressive communication differences. Nurses can promote positive communication and provide appropriate care through supportive action.
23	Winnard et al. (2022). UK, Peer-Reviewed Article	No Specific Time Period (pregnancy, birth-adolescence) Experiences of “being a parent.”	Autistic mothers (*n* = 4) and autistic non-mothers (*n* = 4).	Qualitative Design. Positionality statement. Epistemological position unknown.	Semi-structured interviews, IPA.	1. Parenthood: fun and games 2. Support: giving and receiving 3. Routine and structure 4. Sensory sensitivities 5. Interaction 6. Unique insight.	There are benefits and challenges of being an autistic parent. Autistic-specific skills and traits are associated with strength, resilience, love, nurture, routine, and sensory differences. Positives include parent–child bond, ability to relate to autistic children and being experts by experience to support children's learning, and development. Challenges include battling for support from statutory services, struggling with changes to routine, sensory needs, and multiple demands.

**Table 2.**
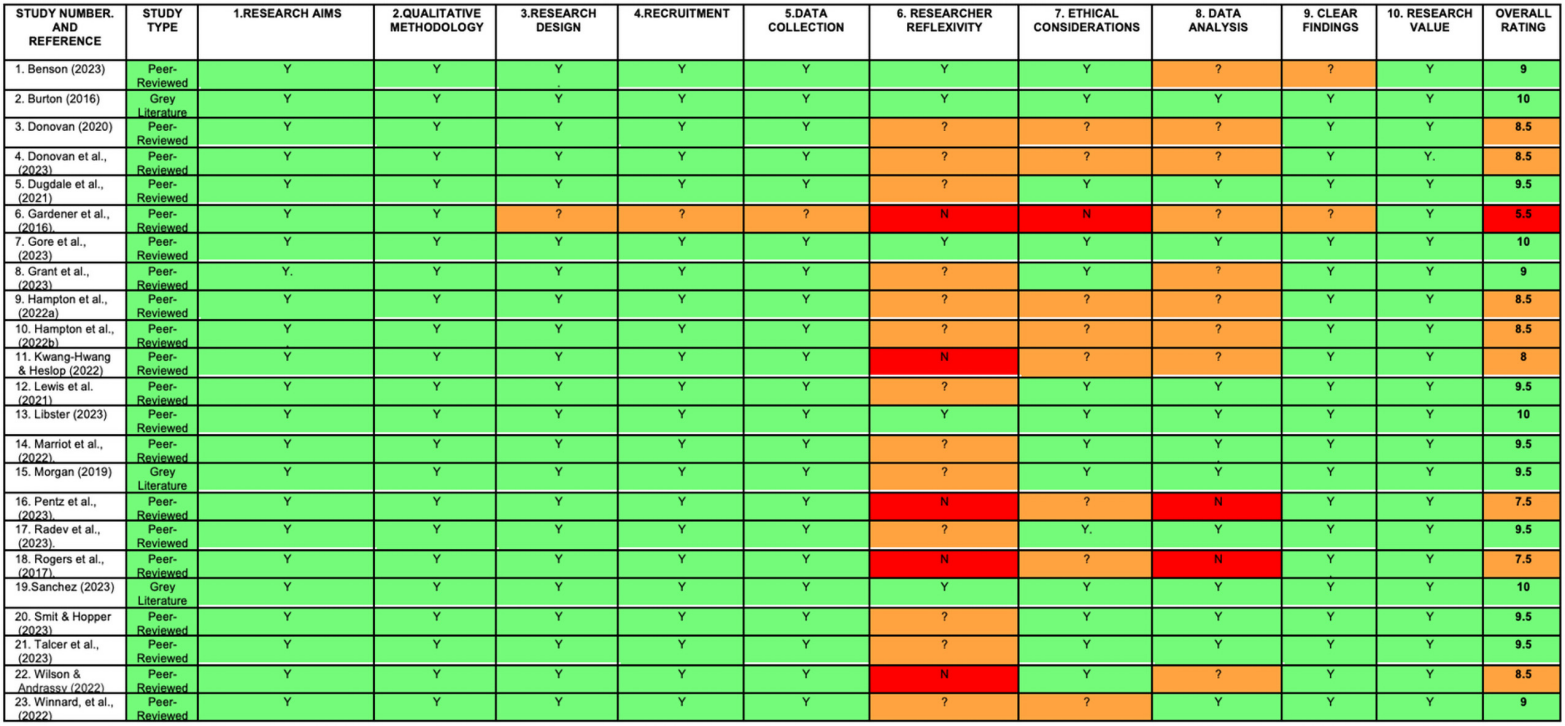
Quality Appraisal Grid.

#### Peer-Reviewed Studies

**Overview.** Twenty peer-reviewed studies from the United Kingdom (12), United States (6) and Australia (2) between 2016 and 2023 were included. Nine were conducted in 2023, five in 2022, three in 2021, one in 2020, one in 2017, and one in 2016.

**Parenting Period.** Studies explored autistic mothers’ experiences from pre-pregnancy to parenting adult children. Ten studies focused specifically on early motherhood, including pregnancy, childbirth, perinatal, post-natal, post-partum, and infancy periods (3, 4, 6, 8, 9, 10, 12, 16, 18, 22). Another (23) had a broad focus, but primarily explored pregnancy. The remaining nine studies explored participants experiences throughout their child's lives from infancy to adulthood (5), or experiences which could occur throughout motherhood (1, 7, 11, 13, 14, 17, 21) or beforehand (20).

**Nature of Experiences.** The nature of autistic mothers’ experiences explored across these periods included: personal childhood, pregnancy, childbirth, bonding, breastfeeding, sensory, employment, health and wellbeing, parenting autistic and non-autistic children, parenting support, interactions with education, social care, pre- and post-natal maternity, acute healthcare, and mental health services including with professionals working within them.

While the nature of experiences varied, twelve studies focused on those specific to early motherhood including pregnancy, childbirth, bonding, breastfeeding, pre-natal and post-natal care, and interactions with healthcare professionals and services throughout this period (4, 5, 6, 8, 9, 10, 12, 16, 18, 21, 22, 23).

The remaining eight studies had a broader focus and explored general experiences during motherhood (5) or multiple experiences simultaneously including general parenting and experiences with education, health, and social services professionals (1, 11, 17), employment (7), mothering autistic children (14) and non-autistic children (13) and mental health, wellbeing, and the influence of childhood trauma (20).

**Sample.** The 20 studies represent first-hand accounts of 361 autistic mothers. Sample sizes ranged from 1 to 141 participants. Nineteen studies had less than 25 participants, one (8) collected data from 141 participants. Fourteen studies included formally diagnosed, self-reported or self-identifying participants (1, 3, 4, 5, 6, 7, 8, 10, 12, 13, 14, 16, 20, 22). Six studies included only formally diagnosed participants (9, 11, 17, 18, 21, 23).

Studies varied with the type, number and quality of sample characteristics collected and reported. No study reliably reported or consistently presented the same demographic data. Fifteen studies reported demographic data in full (3, 4, 5, 6, 7, 9, 11, 12, 13, 14, 16, 17, 18, 20, 21) though some included “prefer not to say” responses. Other studies failed to report demographics for all participants (1, 10, 22). One study (23) did not separate demographics between groups (e.g., autistic mothers and autistic non-mothers) and another (8) did not separate demographics for those who provided quantitative (*n* = 152) versus qualitative responses (*n* = 141). These discrepancies meant an accurate depiction of the 361 participants’ demographics could not be presented.

Commonly reported sample characteristics included: age/age range, formal/self-autism diagnoses, marital and employment status, ethnicity/race, education level and location. Most studies reported whether participants had autistic or neurodivergent children and associated demographics. Data suggests the 361 participants typically represent a subgroup of autistic mothers which were most commonly, white, cis-gendered, highly educated, older aged, late diagnosed, or self-identifying autistic women from higher socio-economic backgrounds with autistic or neurodivergent children.

**Methodologies.** Seventeen studies collated data using semi-structured interviews in various formats including verbal, video, visual and written information. One used focus groups (11), one a qualitative questionnaire (6), and another a mixed method survey (8).

**Involvement.** Most studies utilized participatory research approaches, although the extent of stakeholder involvement varied. Six did not adopt participatory approaches (3, 11, 12, 16, 18, 20). One (6) collated data from a pilot questionnaire initially intended to gather feedback.

**Analyses.** Several analytical approaches were used: one study used Reflexive Thematic Analysis (RTA) (7), eight used Thematic Analysis (TA) (8, 9, 10, 11, 16, 18, 21, 22), six used Interpretative Phenomenological Analysis (IPA) (5, 13, 14, 17, 20, 23), one used Narrative Approach (12) and four used mixed and/or other less common qualitative methods (1, 3, 4, 6).

**Researcher Reflexivity.** Three authors provided detailed reflexive statements, stating their role, influences and epistemological position (1, 7, 13). Eleven authors stated their role but failed to discuss influence or epistemological position (4, 5, 8, 9, 12, 14, 16, 17, 20 21, 23). Six authors failed to do either (3, 6, 10, 11, 17, 22) meaning their assumptions, perspectives and approach to knowledge generation was unclear, reducing rigor and transparency (Creswell & Poth, 2016). All except seven (6, 8, 9, 10, 11, 16, 18) used reflexive strategies to enhance trustworthiness of findings.

**Language.** Studies varied in use of identity or diagnosis-first language. Fourteen used identity-first language “autistic” (1, 3, 4, 5, 7, 8, 9, 10, 13, 14, 17, 20, 21, 22) and six used diagnosis-first language including “with autism spectrum disorder” (3), “with Asperger's syndrome” (6), “with autism spectrum condition” (16, 23), “with high functioning autism spectrum disorder” (18) and “on the autism spectrum” (12).

#### Grey Literature

**Overview.** Three unpublished studies from the UK were included: two doctoral theses (2, 19) and one MSc thesis (15).

**Parenting Period.** The focus ranged from pregnancy to parenting adult children. One (15) focused specifically on early motherhood, another (2) had a mixed focus exploring both early and later motherhood and the other (19) predominantly explored later parenting periods.

**Nature.** Two studies had a relatively specific focus and explored pregnancy and childbirth (15) and interactions with statutory services and professionals (19), while the other (2) explored general parenting including pregnancy and childbirth.

**Sample.** Studies represent first-hand accounts of 268 autistic mothers. Sample size ranged from 7 to 249 participants, two (2, 19) collated data from less than 12 participants and one (15) collated data from 249 participants. Two included formally diagnosed and self-identifying participants (15, 19) while the other (2) only included formally diagnosed. Only one study (2) reported all demographic data collected. One (15) collated data but did not report it, the other (19) only partially reported data. Sample characteristics were similar to peer-reviewed studies (e.g., generally white, cis-gendered, older aged, educated and/or employed autistic women from higher socio-economic backgrounds with autistic/neurodivergent children).

**Methodologies.** Two studies (2, 19) collated data using semi-structured interviews while the other (15) used a mixed-method online survey.

**Involvement.** All studies incorporated meaningful participatory research principles, enhancing the quality, relevance, and impact of the research (Pellicano et al., 2014).

**Analyses.** One study utilized TA (15), one RTA (19), and one IPA (2).

**Researcher Reflexivity.** Two studies explicitly stated and discussed how their epistemological position guided the study, provided reflexive statements, and discussed potential influences (2, 19). The other (15) acknowledged a contextualist epistemological position but failed to discuss the implications of this. All used several reflexive strategies, increasing trustworthiness of findings.

**Language.** Two studies (15, 19) used identity-first language “autistic” and one (2) used diagnosis-first language “with autism spectrum disorder.”

### Quality Appraisal

Quality appraisal should be used meaningfully to inform qualitative evidence synthesis (Garside, 2013; Carroll & Booth, 2015). Confidence in qualitative findings is dependent on the methods used to collect and analyse data and the quality of researchers’ reflexivity (Tod et al., 2022).

The CASP (2020) was used to appraize the quality of all studies (Noyes et al., 2019). Studies were classified and organized into categories from higher to lower quality using the following criteria: 1 point was assigned to “yes,” 0.5 to “cannot tell” and 0 to “no.” Studies scoring 9–10 were deemed “high quality,” 7.5–8.5 “moderate quality,” 6.5–7 “low quality,” and less than 6 “very low quality” (Lewin et al., 2015; Noyes et al., 2019).

Based on current recommendations, all studies were included irrespective of data rating. Limitations were explicitly acknowledged and considered within the synthesis of findings weighted accordingly (Boeije et al., 2023). Results are presented in Table 2, discussed in context of their respective data source thereafter and detailed further in Appendix H.

#### Peer-Reviewed Studies

Eleven studies were deemed “high” quality. Two assigned 10 points (7, 13), six 9.5 points (5, 12, 14, 17, 20, 21), and three 9 points (1, 8, 23*).* Eight were considered “moderate quality.” Five assigned 8.5 points (3, 4, 9, 10, 22), one 8 points (11) and two 7.5 points (16, 18). Only one study was “very low quality,” scoring 5.5 points (6). However, as findings were consistent with the broader qualitative evidence, the study's quality was not considered to significantly impact on the trustworthiness of the synthesized evidence and was included. Overall, the peer-reviewed papers included in this review were of moderate to high quality, with limitations ascribed to poor reporting of epistemologies and limited researcher reflexivity.

#### Grey Literature

All three grey literature studies were deemed “high” quality. Two were assigned 10 points (2, 19), and one assigned 9.5 points (15), increasing their methodological rigor and trustworthiness. Findings were consistent with those reported in peer-reviewed literature.

### Synthesis of Results

Thematic synthesis resulted in 3 analytical themes and 9 subthemes (Figure 3). Additional illustrative quotes are provided in Appendix I.

#### Analytical Theme 1: The Embodied Autistic Experience of Motherhood

All studies explored how autism manifests in motherhood. This theme encapsulates autistic mothers’ strengths and challenges, and the differences between autistic and non-autistic motherhood.

**Subtheme 1.1: “Super Parent Powers.”** Across studies, participants felt that autism positively impacted their experiences of motherhood, enhancing parenting strengths and abilities, and benefitting their children. They “*perceived themselves at an advantage*” (17) viewing autism as “*a super parent power”* (2). For example, describing motherhood as an “*intense fixation” (22), “focus” (17)* and “*special interest”* and reporting a “*craving”* to develop an “*encyclopedic understanding*” to become “*experts”* (8). Participants had “*focused determination”* to understand and meet their children's needs, suggesting that “*having a one-track mind”* enabled them to “*go full force”* to achieve this (22).

Motherhood also “*awakened positive emotions”* (20). Participants were over-excited about motherhood and experienced profound “*joy”* (17) and “*pride”* (20) when “*spending time with”* (23) and “*supporting their children”* (9). They described the “*wins and small things that other parents would take for granted* a *huge celebration”* (5). Motherhood was depicted as relationally rewarding, with the mother–child bond providing “*safety and support”* (13) and “*meaning and purpose”* (2). Participants described “*incredible love”* (20) for their children and valued “*affection, trust and communication in their relationships”* (5). This was ascribed to autistic differences including “*extreme emotional and cognitive empathy”* (13) and *“insider information”* (19).

Most participants suggested autism enhanced their coping abilities and benefitted their children. They were “*able to positively and helpfully impress routine due to their innate strive for structure”* (22) and “*black and white thinking”* (2) provided *“consistency, safety and security in the relationship”* (5). Unique thinking styles led to “*proactive solutions and problem-solving strategies”* (13) including creating birth plans and using strategic masking (8, 13, 14, 19, 21, 23).

**Subtheme 1.2: Motherhood “Amplifies” The “Autisticky Bits.”** This subtheme includes the term “autisticky bits,” a direct quote from a participant in a primary study. The author of the primary study inferred that this related to “how being autistic was a part of them and how their strengths and weaknesses in relation to parenting were, therefore, inherently related to being autistic.”

Across studies, participants noted that intrinsic aspects of motherhood including hormonal changes, overstimulating environments and increased interactions “*amplified”* (5, 9) autistic-specific challenges including sensory, cognitive and communication and emotional difficulties (9). They described motherhood as a “*sensory nightmare”* (15), suggesting sensory challenges were “*intensified”* from pregnancy onwards (8, 21). Compounded by a lack of professional awareness and support, this often led to “*traumatic”* pregnancy, breastfeeding, and birth experiences (2, 4, 12, 15, 17, 20).

The “*relentless nature of motherhood”* (10, 14), including increased social, sensory, and cognitive demands, was also challenging. Participants reported difficulties adapting to changes and tolerating uncertainty as it opposed innate “*preferences for routine”* (13) “*predictability”* (9), and “*structure”* (23). They also struggled to understand and cope with “*social dynamics and situations”* due to communication differences (6, 13, 19). Moreover, being excluded, misunderstood, or misinterpreted by others including parents and professionals evoked stress, fear, guilt and frustration and self-critical thoughts about “*not being good enough*,” *“failing in life”* (5,19) and “*failing to meet social expectations of parents”* (15).

“*Extreme empathy”* presented more intensely during autistic motherhood, with participants “*experiencing their children*'*s emotions as their own”* (20) and feeling “*undue stress and anxiety”* (5), from striving for “*perfectionism”* (7). To cope with challenges, many participants sacrificed their needs, relationships, education, and careers and used masking to appear “*normal”* and “*fit in”* (5, 19).

***Subtheme 1.3: Similar Yet Different.*** Studies highlighted the similarities and differences between autistic and non-autistic motherhood. Most participants felt “*different”* (2) to non-autistic mothers, like they “*did not fit the normal moms club”* (5). Some discussed having different life experiences, noting adverse experiences related to autism (6, 14, 20). They also acknowledged shared experiences, highlighting intrinsic, universal aspects of motherhood including bodily changes, childbirth, and infant feeding (2, 9). Studies recognized differences between participants’ experiences, with some reporting positive sensory experiences or delayed bonding, while the majority did not (8, 21).

#### Theme 2: Navigating the Non-Autistic World as an “Other” Mother

All studies illustrated how participants’ interactions with others in different social contexts shaped their experiences of motherhood, and “*professionals held the power to either positively or negatively impact participants’ mental health through acceptance and awareness (or lack thereof)”* (20).This theme highlights the interaction between autistic mothers and varying social contexts, offering insight into participants encounters with professionals, services, settings, systems, and society and the impact.

**Subtheme 2.1: “Mothered”; Systemic, Structural, And Societal Othering.** Almost all participants reported adverse or traumatic experiences from feeling othered by professionals and their practices which precipitated distress. Studies suggested that this occurred most frequently during interactions with health, education, and social care professionals.

Participants discussed feeling overpowered, explaining how professionals exerted expertize and authority over them by making decisions on their behalf and expecting them to “*comply automatically*” (1, 4, 19). This included failing to seek consent before “*touching them”* (16), “*turning up at their home”* (1), or adapting communication throughout critical moments including childbirth (12). Participants also depicted feeling policed and pathologized by professionals, explaining how “*neuro-normative parenting ideologies*” were “*imposed”* on them (1) and how they, or their parenting was “*discouraged”* (18) or “*rejected”* (17). Professionals reportedly had a “*one size fits all”* (22) approach, mistreating and discriminating against participants when they did not fit the “*neurotypical mould”* (21). This included being “*told off”* (22), “*bullied*,” (2, 18) “*dismissed”* (4, 5, 6, 7, 8, 9, 10, 12, 15, 17, 19), “*intimidated*,” *(18, 20) “threatened”* (15), and “*ignored”* (13, 18, 22). Some received explicit comments: “*I’ve been asked by a couple of the midwives how I think I can be a mum if I’m autistic*” (9).

Participants reported how professionals perceived them, their differences and parenting as “*dysfunctional”* (18), “*suspicious”* (1) or misinterpreted them for “*mental instability”* (3). Consequently, some participants were “*forced to undergo parenting”* or “*capacity assessments*” (1, 15, 19), referred to safeguarding and social services or had children removed (1, 2, 19). Participants’ interactions and experiences with professionals were concluded as “*traumatizing”* (2, 4, 12, 15, 17, 20) and led to “*internalized stigma”* (2), “*perfectionism”* (7, 20), “*self-doubt”* (2) and “*anxiety”* (1, 3, 4, 9, 10, 12, 15, 18, 20).

Participants described feeling othered by “*hostile”* (1) environments and when accessing support, explaining how they were expected to “*fit”* hospital settings and support groups for non-autistic mothers (1, 8, 16, 21). Hospitals were experienced negatively by almost all participants in early motherhood, with reports of requests for adjustments or adaptions being denied, leading to “*traumatic” “pregnancy”* (21) “*birth”* (12) “*breastfeeding”* (8) experiences and “*cognitive, sensory and information overload*” (20). Participants reported that parenting information, resources, and advice predominantly targeted non-autistic mothers, reinforcing a sense of othering (6, 4, 21).

Participants described othering when attempting to access health and social services for support for themselves or their children, explaining that they “*fell through the gaps of existing services*,” (19) and felt “*marooned at sea”* (14), resulting in an ongoing “*battle”* (17) and “*fight”* (2, 5, 23) against systems. Most were denied help, “*prescribed neuro-normative solutions”* (1) to change themselves, improve their parenting (2, 4, 15, 19) or had their parenting abilities questioned, which was suggested to “*locate problems within autistic mothers”* (1) and evoked beliefs about “*having something wrong with them*” (2). Most participants and their children remained unsupported and found it “*exhausting*” to “*battle systems”* (1, 17).

**Subtheme 2.2: “Rare Positive Experiences.”** Studies indicated that positive interactions and experiences with professionals, services, systems, and others occurred infrequently, predominantly in early motherhood. Participants specified the “*one*,” or “*few*” professionals involved, considering themselves “*lucky”* (13, 18). Positive experiences were attributed to professionals being “*proactive”* in understanding and meeting participants’ autism-specific needs including collaborative planning and adapting communication and environments (6, 10, 16). Participants valued these professionals’ “*kindness”* (10) “*empathic”* (2), “*non-judgmental”* (17) and “*accepting”* (2) approach, and “*continuity of care and consistency”* from services, which increased trust and met autism-specific needs (10, 16). Many participants reflected positively on their social networks providing advice and support (2, 5, 9 10, 19) and some felt “*empowered”* from “*connecting with*,” and “*sharing experiences and advice”* with other autistic mothers (21).

**Subtheme 2.3: Sense Making, Surviving the Storm and Empowering a New Norm.** Across studies, participants attempted to make sense of their adverse, othering experiences. All studies suggested communication differences and poor understanding about autism led to professionals misinterpreting participants’ behaviors (4, 5, 9, 17, 22). This was indicated by professionals’ *“offensive, deficit-based, stereotyped, and outdated”* (17) and “*medicalized”* (18) views and language about autism and women. Others reflected on systemic problems, suggesting dominant, historical discourses about mothers and disability underpinned the structure of services and systems, contributing to them feeling othered and excluded (2, 18, 19).

Collectively, participants depicted striving to survive feeling othered and discriminated against. Many used coping strategies including “*masking”* (5, 7, 13, 5, 19, 21) and “*picking their battles”* (17) when interacting with professionals, or sought advice from their social networks or other autistic mothers (2, 5, 9, 10, 19). Some expressed determination to challenge or resist normative parenting ideologies and interventions imposed by professionals and society, advocating for parenting their way (1, 2, 21). They described themselves and their parenting as “*different not deficient”* (19) suggesting autism did not impact their abilities. Others expressed empowering, self-affirming statements about their strengths, highlighted the mismatch between their views and professionals, while anticipating they would “*face judgement”* for “*going against the norm*” and “*mothering their way*” (5, 19).

Across studies, there was consensus that greater societal awareness about autistic mothers, professional training and autism-specific services were needed to support them and their children, reduce othering and increase acceptance.

#### Theme 3: Recalibrating Identities

Participants experienced multiple shifts in their identities during motherhood. This theme encapsulates their experiences of understanding and adapting to their autistic and maternal identities including both positive and challenging aspects.

**Subtheme 3.1: Mixed Feelings About Becoming a Mother.** Participants had mixed experiences of adjusting to their maternal identity and roles. For some, it was a positive journey *of “self-growth” (2) and “self-acceptance”* (5). They felt “*confident in their mothering abilities*” (19), reporting increased “*tolerance”* (2) and *“flexibility”* (5) to adapt to change which evoked feelings *of “pride”* (20) and *“achievement”* (23). Other participants reported challenges processing “*the loss of their identities and lives”* (20) before motherhood and coping with the changes and challenges they experienced. They often struggled to “*find a balance”* between their maternal identity and other social roles, suggesting their lives were defined by striving to be “*seen and understood as individuals”* (22), “*good mothers”* (6) and *“competent parents”* (19).

**Subtheme 3.2: Understanding and Integrating Autism.** Most participants self-identified or were diagnosed as autistic during motherhood. For some, this was a *“validating”* (5), *“lifesaving”* (2) “*lightbulb moment”* (17) which “*increased understanding, self-compassion, and self-acceptance”* (5). It also helped them to “*make sense of and reframe their earlier life experiences in a new light”* (17), often attributing challenges to autism, poor support, and challenging environments rather than *“personal failures”* or “*inadequacies”* (19, 21). They also better understood how autism influenced their strengths and challenges in motherhood (2).

Others reported difficulties in understanding and adjusting to their autistic identity, describing it as a cognitively demanding, emotional process that evoked feelings of “*guilt”* (5, 7) and “*self-blame”* (8). The timing of their diagnosis was challenging in context of adjusting to motherhood, “*competing cognitive demands”* and *“a lack of professional support”* (7, 21). Participants suggested that “*understanding their autistic identity earlier”* could have led to support and reduced challenges in motherhood and their lives (7, 13, 14).

**Subtheme 3.3: Sharing Autistic Identities.** Participants shared their fears and hopes of disclosing autism in motherhood. Most “*feared disclosing to professionals”* (6) anticipating they would be stigmatized, treated negatively or questioned due to the “*invisibility”* of autism (7), or perceived as “*risks”* to their children (21). They felt lack of societal awareness and understanding about maternal autism increased risk of negative repercussions. Some hoped disclosure would “*raise awareness of autistic mothers*” (7).

Most participants reported negative experiences when disclosing to professionals, suggesting “*outing diagnosis was more harmful than helpful”* (15). They explained that professionals instantly changed by making negative comments, using “*patronizing”* (5), “*infantilizing”* (20), and “*offensive”* (12) language and *“challenging their diagnoses”* (17, 19) based on “*traditional views of autistic boys”* (9). Participants had similar experiences when disclosing children's diagnoses (1, 11, 14, 17, 19). Many “*regretted”* (19) disclosing, felt “*unable to trust professionals”* (9, 13) and described feeling “*mislead and lied to”* when harmful responses or negative repercussions followed (17). Other participants disclosures were *“ignored”* or *“overlooked”* (7, 10, 12, 16) resulting in unmet needs (1, 11, 14, 15).

Positive experiences of disclosure were infrequent, occurring mostly in early motherhood. Participants discussed how midwives viewed them as “*experts*,” used “*individualized approaches”* (9), made “*environmental adaptions”* (21), or “*went above and beyond to do their homework”* (15) to understand and support them. Most found disclosing to social networks positive (2, 8, 9, 10, 14, 18, 22), reporting acceptance, “*practical and emotional support”* (10), and strengthened relationships, particularly with neurodivergent children (5, 11, 13, 14, 15).

## Discussion

This review aimed to synthesize existing qualitative evidence on autistic mothers’ lived experiences of motherhood to address gaps in extant literature, and inform future research and practice (Grypdonck, 2006; Noyes et al., 2023). Three themes were developed, representing the collective experiences and perspectives of 629 autistic mothers from 23 primary studies: “The Embodied Autistic Experience of Motherhood,” “Navigating the Non-Autistic World as An ‘Other’ Mother,” and “Recalibrating Identities.” Each theme elucidates different facets of their experiences. Together, themes highlight a complex process, with interactions and at times conflicts between experiences of motherhood, autism, identity, and socio-cultural contexts.

### Theme 1: The Embodied Autistic Experience of Motherhood

Theme one reveals how autism influences, and is influenced by motherhood, presenting unique strengths and challenges for autistic mothers and positive and difficult parenting experiences. It highlights similarities and differences between autistic and non-autistic motherhood, while acknowledging that autistic mothers’ experiences differ based on their unique identities and contexts. For example, autistic mothers perceived autism as a “super parent power” (Burton, 2016) reporting strengths including specialized interests, focused determination, extreme empathy, insider insight, and creative, structured, and solution-focused thinking and problem-solving. These strengths enhanced their parenting abilities and coping, giving them an “advantage” (Sanchez, 2023; Winnard et al., 2022) over non-autistic mothers, particularly those with neurodivergent children.

This aligns with previous research, where “the autistic advantage” is increasingly used to capture autistic peoples’ strengths (Russell et al., 2019a, 2019b). However, this review reveals new insight into how these strengths manifest throughout motherhood. For example, by enhancing autistic mothers’ ability to attune to their children and provide consistency, which are considered essential factors for children's physical, communicative, social, emotional, and cognitive development (Ainsworth et al., 1974; Bell & Ainsworth, 1972; Grolnick et al., 2014) and are related to positive child and family outcomes (Gattis et al., 2022).

Findings suggest neuro-affirming language and approaches which leverage and recognize autistic mothers’ strengths are needed, consistent with previous research, and professional guidelines for working with autistic parents and families (BPS, 2021; Department of Health and Social Care, 2022; HM Government, 2021; Kwang-Hwang & Heslop, 2022; National Institute for Health & Care Excellence, 2021). However, given the challenges reported, this review indicates a need to address professionals’ adherence to these guidelines.

Theme one also highlights how motherhood amplified the intrinsic challenges of autism, as reported by diverse groups of autistic people across contexts, including social, cognitive, and sensory differences (NAS, 2024). However, findings expand current understanding, showing how these challenges manifest in the context of motherhood. For example, autistic mothers experienced motherhood as a “sensory nightmare” (Morgan, 2019) and reported difficulties coping with the ever-changing, cognitively, and socially demanding, relentless nature of motherhood. Compounded by neuro-normative parenting ideologies frequently imposed by professionals and society, and poor professional understanding and support, these challenges increased distress, resulting in many autistic mothers sacrificing their needs to prioritize their children.

These findings are consistent with the experiences of other autistic groups (Gosling et al., 2023; Turnock et al., 2022) and reinforce previous recommendations that greater awareness and support is needed, particularly for women (Cook et al., 2024; Milner et al., 2019). Findings also indicated autistic mothers’ experiences differ to non-autistic mothers, reflect their unique identities, life experiences and socio-cultural contexts, and include positive sensory experiences, consistent with broader autism literature (Lord et al., 2018; NAS, 2024). This suggests systemic changes may be needed to provide services specific to autistic mothers’ individual needs.

### Theme 2: Navigating the Non-Autistic World as an “Other” Mother

Theme two illustrates the power professionals had in shaping and defining autistic mothers’ experiences of motherhood and impacting their psychological wellbeing (Smit & Hopper, 2023). It depicted the pervasive sense of othering autistic mothers experienced when interacting with professionals, services, systems, and society throughout motherhood and the detrimental impact this had, with positive experiences infrequently reported. This aligns with research arising from the neurodiversity paradigm which posits that socially constructed barriers negatively impact neurodivergent people. Such adverse experiences increase the prevalence of psychological challenges in the autistic population, with autistic women considered at higher risk (D’Mello et al., 2022; Lai et al., 2019; Yau et al., 2023).

However, findings suggest autistic mothers may be increasingly vulnerable due to contact with professionals throughout their children's lives who reportedly impose gendered expectations and normative parenting ideologies upon them (Benson, 2023; Radev et al., 2023; Sanchez, 2023). This includes comparisons to non-autistic mothers, negative language, behaviors, and comments related to autism and motherhood which were commonly concluded as “traumatizing” (Benson, 2023; Burton, 2016; Donovan, 2020; Lewis et al., 2021; Morgan, 2019; Rogers et al., 2017).

The second theme also depicted participants’ reflexive abilities and desire for change through their attempts to conceptualize their experiences and survive feeling discriminated against by locating the challenges with professionals and society, rather than themselves. They reframed their autistic differences and unique parenting styles as “different not deficient” (Sanchez, 2023) suggesting being autistic did not negatively impact their abilities. This collective sense of empowerment indicated a rejection of conformity and dominant, historical discourses for a more accepting and affirming perspective, consistent with the neurodiversity paradigm (Pellicano & Den-Houting, 2021), illustrating the strengths of this group and highlighting the importance of integrating their first-hand experiential perspectives with recommendations for change in research and practice, particularly given the historical under-representation of this group in research and society (Milton, 2014; Pohl et al., 2020).

### Theme 3: Recalibrating Identities

The final theme highlights the multiple identity shifts autistic mothers experienced. This includes positives and challenges of recalibrating their personal identities to integrate maternal and autistic identities, with most learning they were autistic during motherhood. This was a mixed experience, with some autistic mothers describing a journey of self-discovery, personal growth and acceptance, and others depicting a sense of unambiguous loss and grief. Similar experiences are commonly reported by autistic adults diagnosed in adulthood (Corden et al., 2021). However, research suggests autistic women face increased challenges because dominant autism discourses can act to police normative gender expectations, negatively impacting the adjustment and acceptance process (Moore et al., 2022; Turnock et al., 2022).

Furthermore, findings suggest autistic mothers have unique experiences related to the complex process and impact of simultaneously adjusting to motherhood and their autistic identities, exacerbated by harmful responses relating to gender and role norms and expectations when disclosing autism. This reinforces the need for greater societal awareness and understanding of the additional challenges experienced by this group, and supports recommendations for improved, timely diagnostic processes and post-diagnostic support for autistic women, particularly mothers (Cook et al., 2024; Gosling et al., 2023; Kelly et al., 2022; Milner et al., 2019).

## Strengths

This novel review amalgamated existing peer-reviewed research and grey literature on autistic mother's experiences throughout motherhood, broadening and updating understanding from previous syntheses (Ferrara et al., 2023; Grant et al., 2022; McDonnell & DeLucia, 2021; Samuel et al., 2022; Stuart & Kitson-Reynolds, 2024; Thom-Jones et al., 2024; Turner, 2021). Several databases were searched to capture all available qualitative data and including grey literature increased variation.

Thematic synthesis allowed the reviewer to “go beyond” individual study findings to integrate autistic mothers’ experiences across various stages of motherhood and socio-cultural contexts. This provided a holistic view of the topic, while presenting novel insight into this population's experiences. Detailed study characteristics helped preserve the context of primary studies, allowing transferability of findings across time and contexts (Thomas & Harden, 2008).

Several reflexive strategies were used alongside the systematic process to enhance methodological rigor (Noyes et al., 2023). The reviewer kept a reflexive journal to note their perspectives and potential influences throughout the review (Olmos-Vega et al., 2022) and documented this within a reflexive statement to increase transparency and coherence (Yardley, 2000). They engaged in reflexive supervision with the second reviewer to make decisions around the reviews’ methodology and thematic structure, enhancing commitment and rigor (Bieler et al., 2021; Yardley, 2000). The reviewer extensively engaged with research on the topic, and broader autism literature, enabling them to contextualize findings while honoring participants voices and their subjective interpretation, and establish impact and importance (Thomas & Harden, 2008). Illustrative quotes from contributing studies were included and embedded into findings to preserve their context and demonstrate how themes reflected participants voices rather than “emerging” from the data, further increasing transparency and rigor (Holmes, 2020; Yardley, 2000).

## Limitations

Limitations relating to primary studies’ characteristics and methodological rigor must be acknowledged and used to exercise caution about this review's conclusions. While 23 studies of moderate to high quality were included, many researchers failed to state their epistemology and adequately discuss their potential influences. Most used reflexive strategies to counter this, however, researcher reflexivity and clear epistemological positioning increases transparency, trustworthiness, and methodological rigor of qualitative research (Creswell & Poth, 2016; Yardley, 2000) and the relevancy and impact of autism research (Kaplan-Kahn & Caplan, 2023). Future studies should prioritize ensuring these aspects of rigor are achieved. Similarly, many studies failed to detail the ethical implications of researching a potentially distressing subject, highlighting a need for future research to consider and document how participants were protected from harm.

Across studies there was significant variation in the type, number and quality of sample characteristics collected and reported. None reliably reported or presented the same demographic data. Most failed to consider how aspects of difference may impact autistic mothers’ experiences. This posed challenges accurately reporting the demographics of participants represented in this review and situating their experiences in relevant contexts as required (Thomas & Harden, 2008). The data reported and synthesized suggests this review predominantly reflects the experiences of white, cis-gendered, highly educated, older aged, late diagnosed, or self-identifying autistic women, from higher socio-economic backgrounds across the UK, the US, and Australia, with neurodivergent children.

Almost all studies acknowledged homogenous samples as a limitation, suggesting further research with autistic mothers of younger ages, from diverse socio-economic and cultural backgrounds, with co-occurring differences is needed to increase representativeness and clinical relevance of findings. This is particularly important in context of the limited research in the area, and the high prevalence of co-occurring differences amongst this population (Lai et al., 2019). Moreover, cultural, and contextual factors can influence the expression of autistic differences, diagnostic processes, and the availability and nature of support services (DeLeeuw et al., 2020). Similarly, motherhood, and the experiences of women in general may differ across cultures and contexts (O’Reilly, 2014). These factors are likely to influence autistic mothers’ experiences, highlighting a need for increased sample variation and cross-cultural research on this topic.

Across studies, there was marked variation in the language used to refer to autistic mothers. Studies using diagnosis-first language raise concerns about whether researchers prioritized participant preferences, and the extent to which findings accurately reflect participants voices, particularly when studies failed to reflect on their position, bias, or use participatory approaches. Diagnosis-first language can perpetuate autism stigma, suggesting a need for researchers to adhere to relevant autism research guidelines to improve research (Kaplan-Kahn & Caplan, 2023; Monk et al., 2022).

Similarly, there was variation in the way motherhood and parenting were defined, but not all studies captured this in their titles. Despite the comprehensive search strategy, it is possible that studies omitting these terms in titles or abstracts remain unidentified. Defining and standardizing terminology is essential to ensure all available evidence is included in future reviews. Timing of this review may also mean relevant emerging studies were excluded. Further systematic reviews will help to continually update understanding. This may be important given the changing context of studies exploring autistic mothers’ experiences in recent years, moving away from early motherhood to exploring specific, predominantly adverse experiences throughout their children lives such as interactions with statutory services (Benson, 2023; Kwang-Hwang & Heslop, 2022), employment experiences (Gore et al., 2024) and parental wellbeing and childhood influences (Smit & Hopper, 2023).

This review included grey literature sources to increase the body of literature used to produce a meaningful synthesis (Godin et al., 2015). Autistic mothers commonly, and increasingly, discuss their experiences in online forums, blogs, magazine articles, and books (Grace, 2021; Grant, 2015; Litchman et al., 2019; Prince, 2010). While these sources were excluded from the current review due to concerns around methodological rigor, this potentially indicates a need to increase research opportunities for autistic mothers to share experiences, given they remain an under-researched, under-represented group (Thom-Jones et al., 2024). Utilizing qualitative, participatory approaches recommended for, and by autistic communities may help overcome challenges such as fears around interacting with professionals, as identified in this review.

## Clinical and Research Implications

This review's findings support recommendations for professional training, best practice guidelines, improved diagnostic processes, individually tailored support, and specific support services for different stages of autistic motherhood. They also highlight a need to understand the barriers to translating research recommendations to practice, given all included studies have recommended changes to prevent negative experiences and increase support for autistic mothers, yet findings suggest mothers continue to endure challenges without support. Further research around specific adverse experiences could broaden understanding about the impact and help identify contributing factors to prevent harm and inform support.

Overall, this review stresses the importance of increasing awareness and support for autistic mothers, addressing barriers to implementing previous research recommendations, and preventing harm caused by poor understanding and discriminatory practices through changes to best practice guidelines for professionals working with this group.

## Conclusion

Autistic mothers report having unique strengths and prioritizing their children above all. However, they experience challenges related to the interaction between autism and innate aspects of motherhood. Further complexities arise from becoming aware of, or being diagnosed as autistic during motherhood, and from adjusting to, and sharing their autistic identities with others. Moreover, their experiences of motherhood are largely colored by adverse experiences related to systemic, structural, and societal othering, leading to increased psychological challenges. Specifically, from feeling policed, pathologized, and overpowered by professionals. While autistic mothers show increased resilience and determination to mitigate and cope with the autistic and identity-specific challenges they experience throughout motherhood, their ability to cope with the pervasive sense of othering they encounter during interactions with professionals and in society is limited and impacts their psychological wellbeing. Further research, professional training, systemic changes, and societal awareness are urgently needed to increase understanding, prevent psychological harm, and inform support for this group.

## Supplemental Material

sj-docx-1-dli-10.1177_23969415251343850 - Supplemental material for The Lived Experiences of Autistic Mothers: A Systematic Review and Thematic Synthesis of Qualitative Evidence Supplemental material, sj-docx-1-dli-10.1177_23969415251343850 for The Lived Experiences of Autistic Mothers: A Systematic Review and Thematic Synthesis of Qualitative Evidence by Deanne Christie Lockington and Fiona Gullon-Scott in Autism & Developmental Language Impairments
