# Supplementary material for: The Lived Experiences of Autistic Mothers: A Systematic Review and Thematic Synthesis of Qualitative Evidence
Source: Autism Dev Lang Impair. 2025 Jul 7;10:23969415251343850. doi: 10.1177/23969415251343850 (PMC12246537; doi:10.1177/23969415251343850)
Supplement: sj-docx-1-dli-10.1177_23969415251343850 - Supplemental material for The Lived Experiences of Autistic Mothers: A Systematic Review and Thematic Synthesis of Qualitative Evidence [file sj-docx-1-dli-10.1177_23969415251343850.docx]

**Appendices**

**Appendix A - Population, Interest and Context Framework**

| **Population (P)** | Autistic Mothers |
| --- | --- |
| **Interest (I)** | Lived Experiences of Motherhood |
| **Context (Co)** | Existing Qualitative Research on First-Hand Accounts |

**Appendix B - Inclusion and Exclusion Criteria**

| **FACTOR** | **INCLUSION** | **EXCLUSION** |
| --- | --- | --- |
| **SAMPLE** | - Studies whose participants are mothers, with a clinical or self-diagnosis of autism. - Studies whose participants are mothers to at least one child (including expectant, adoptive, surrogate, stepmothers and/or primary carers who identify as mothers). - Studies which include autistic and non-autistic mothers, autistic non-mothers, or autistic fathers where qualitative data is demarcated between participant groups. | - Studies whose participants are non-autistic mothers (e.g., women without a clinical or self-diagnosis of autism). - Studies whose participants are autistic non-mothers (e.g., are not expectant, adoptive, surrogate, stepmothers and/or primary carers who identify as mothers). - Studies which include autistic and non-autistic mothers, autistic non-mothers, or autistic fathers where qualitative data cannot be demarcated between participant groups. |
| **MAIN FOCUS OF STUDY** | - Studies which include self-reported data from autistic mothers about any experience related to motherhood and/or parenting. | - Studies reporting data of the experiences of other family members and/or caregivers (e.g., fathers) and/or professionals which do not include self-reported data from autistic mothers. - Studies reporting information which does not relate to experiences of motherhood or parenting. |
| **DESIGN** | - Qualitative studies (e.g., interviews, focus groups, qualitative surveys, case studies) and analyses (e.g., Interpretative Phenomenological, Thematic, Content, Discourse or Narrative Analyses, Grounded Theory, other qualitative methods) with self-reported qualitative data from autistic mothers about motherhood/parenting experiences. - Mixed-method studies with self-reported qualitative data from autistic mothers about motherhood/parenting experiences where qualitative data is clearly demarcated. | - Quantitative design studies. - Qualitative studies which do not report first hand experiential data from autistic mothers (e.g., observational studies). - Mixed-method studies with self-reported qualitative data from autistic mothers about motherhood/parenting experiences where qualitative data is not clearly demarcated. |
| **RESEARCH TYPE** | - Published peer-reviewed research (e.g., journal articles, dissertation, theses and/or manuscripts and conference proceedings). - Grey literature which has a degree of scientific and methodological rigour and merit via a review process (e.g., university review) including all thesis and/or dissertations. | - Grey literature which does not have a degree of scientific and methodological rigour and has not been approved via a review process (e.g., university review). |
| **DATE** | - Publication date after 1911. | - Publication date before 1911. |
| **LANGUAGE** | - Studies available in English. | - Studies not available in English |

**Appendix C - Search Terms**

The following categories and keywords were used for the systematic search:

| **CATEGORY** | **KEYWORDS** |
| --- | --- |
| **MOTHERHOOD** | Mother* OR Female* Parent* OR Perinatal OR postnatal OR postpartum OR pregnan* OR childbearing |
| **AUTISM** | Autis* OR Asperg* OR ASD OR ASC OR Neurodevelop* OR Neurodiver* |

**Appendix D - Data Collection Form**

Study Information

Authors:

Year of Publication:

Title:

Country of Origin:

Type of Study:

Parenting Period:

Nature of Experiences:

Participant Information

Population:

Sample size:

Sampling method:

Recruitment:

Eligibility Criteria:

Sample Characteristics:

Methods

Design:

Epistemology:

Researcher Reflexivity:

Data Collection:

Data Analysis:

Key Findings:

Key Themes:

Summary:

**Appendix E – Critical Appraisal Skills Programme (CASP) Checklist for Qualitative Research**


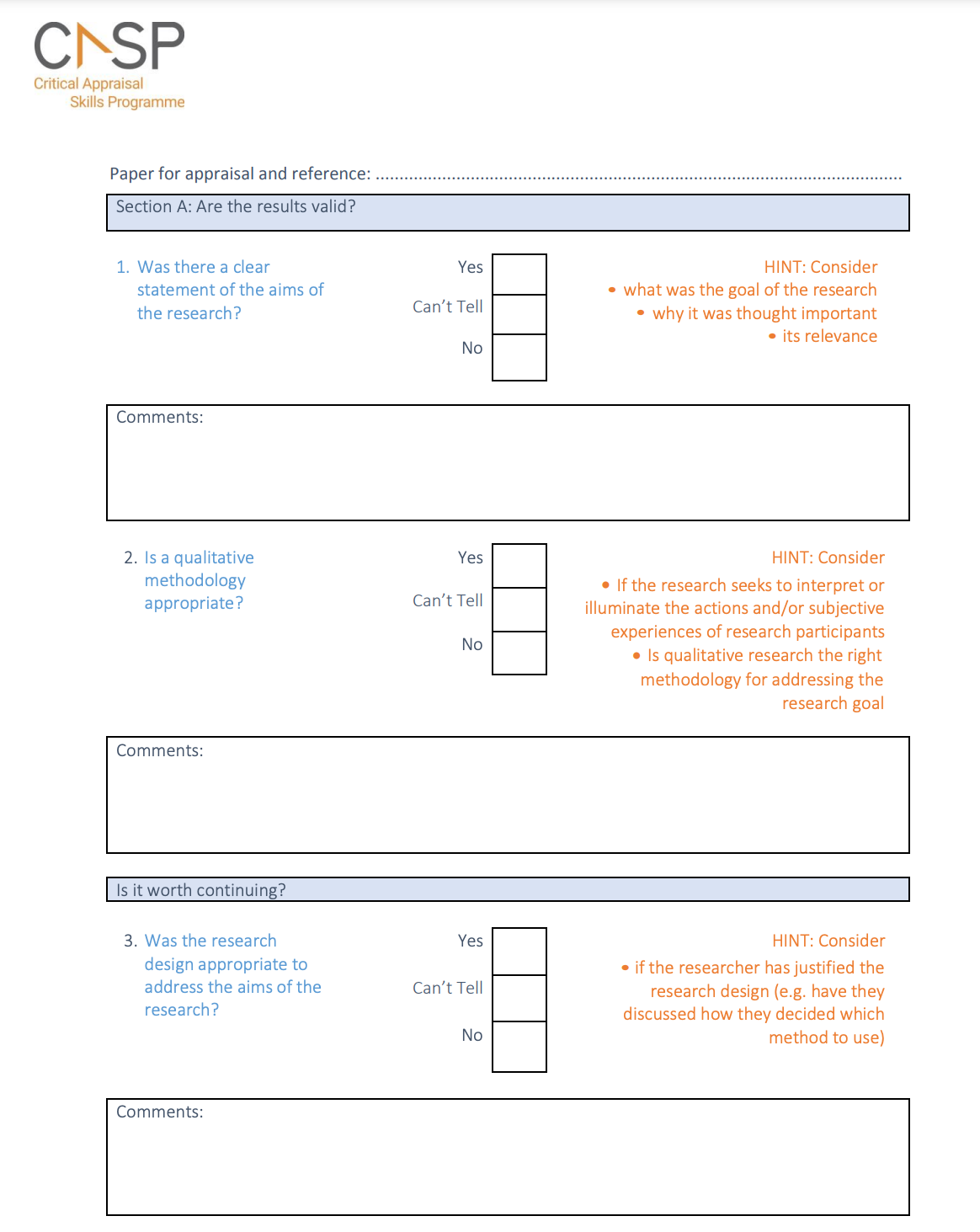


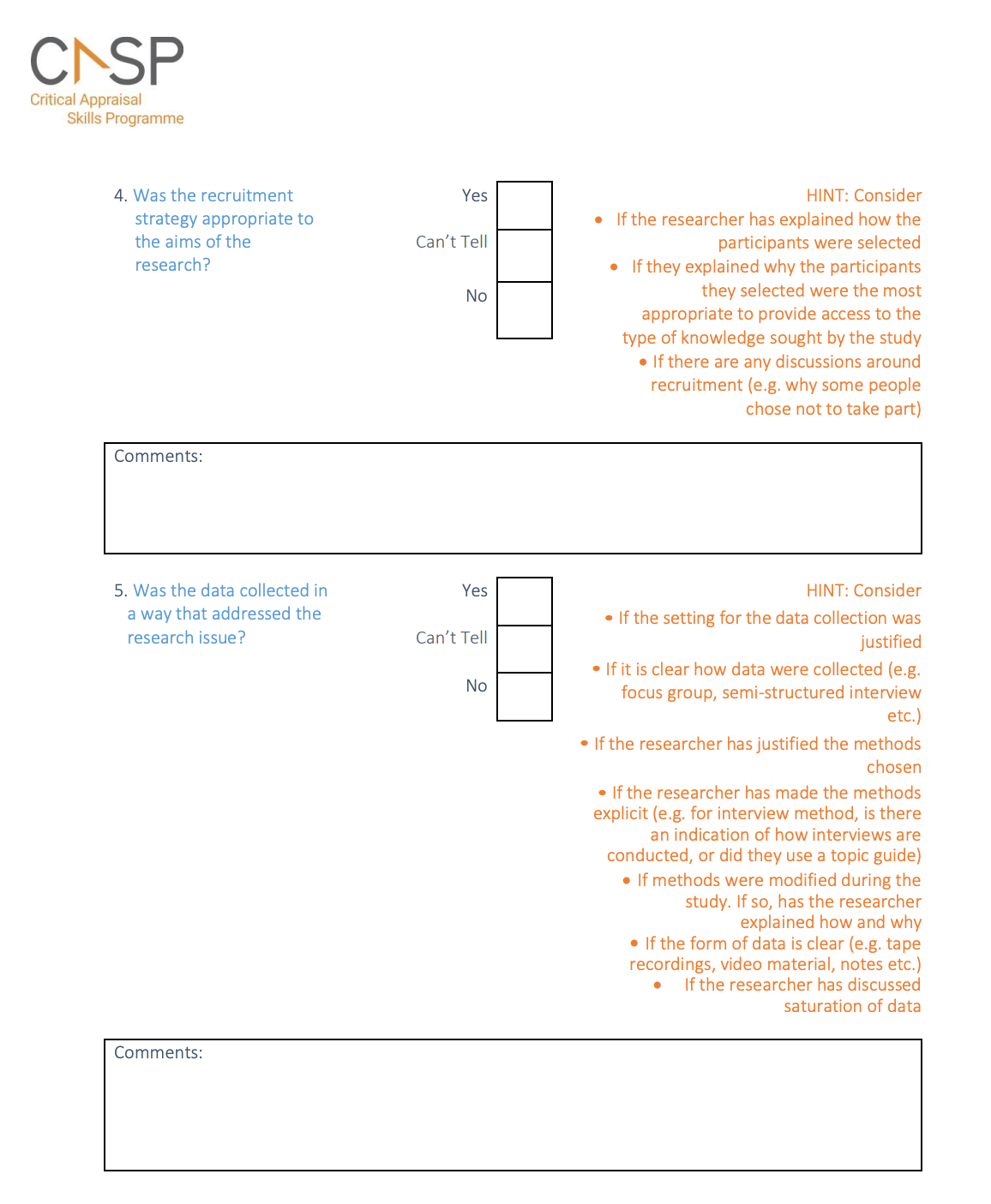

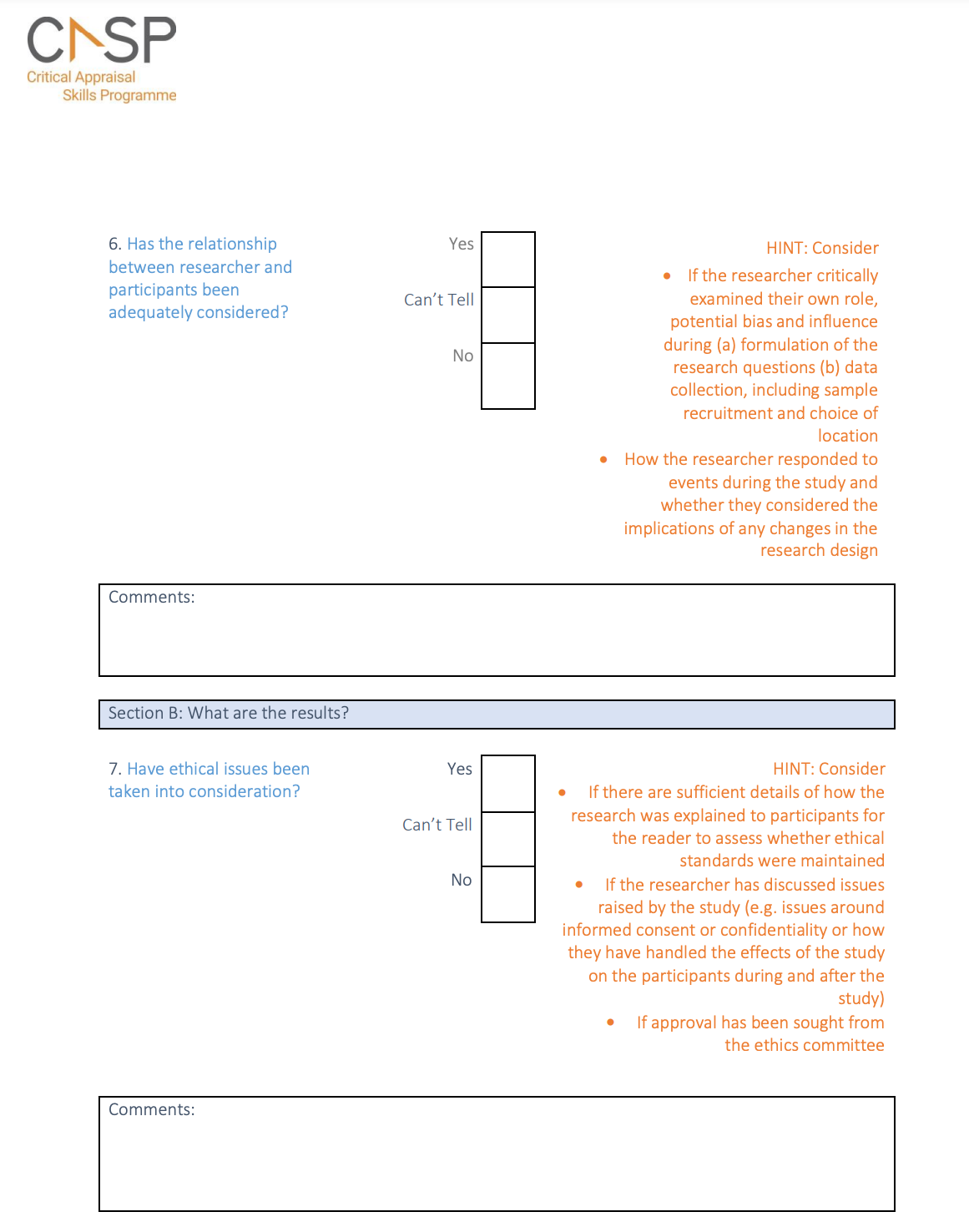

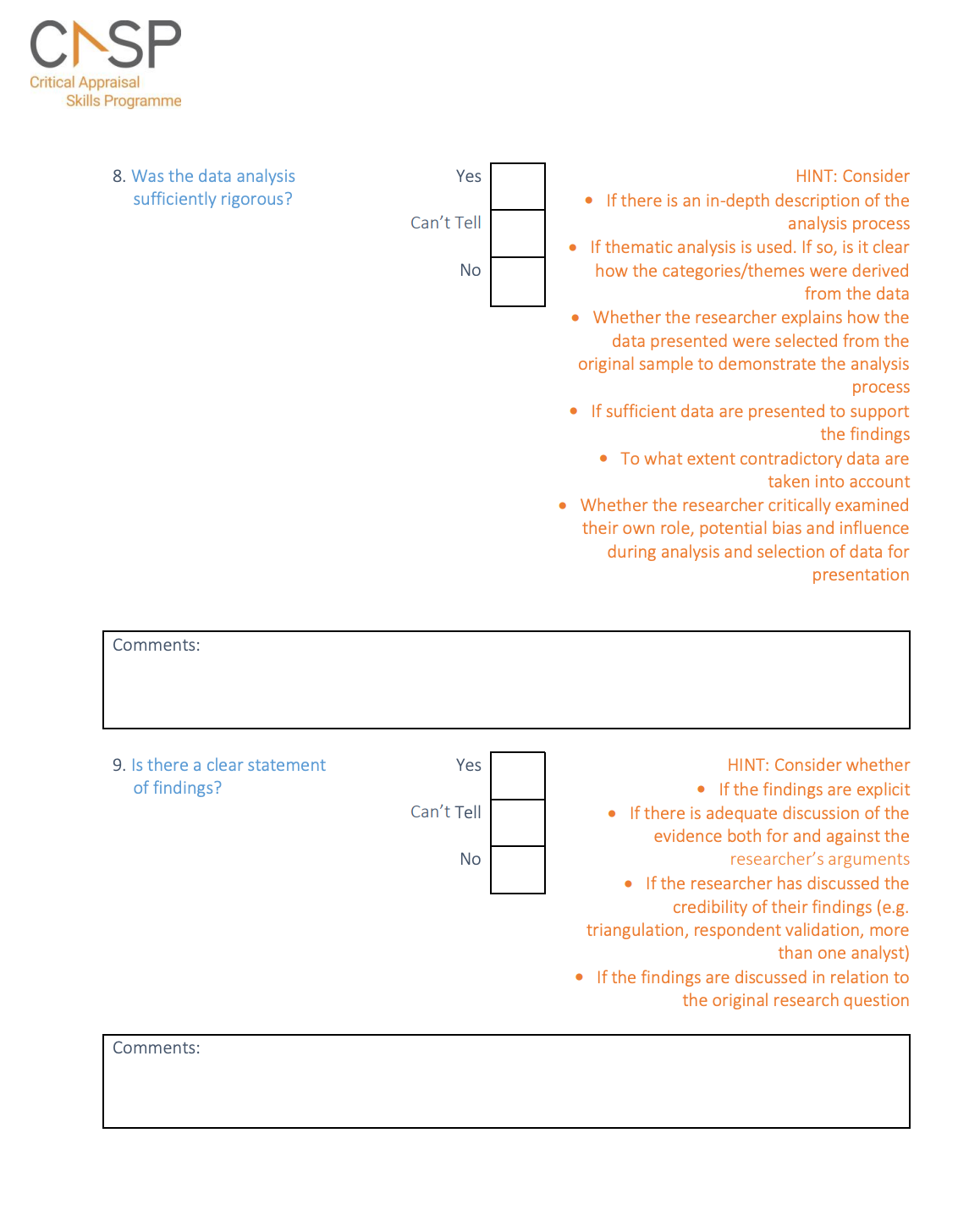


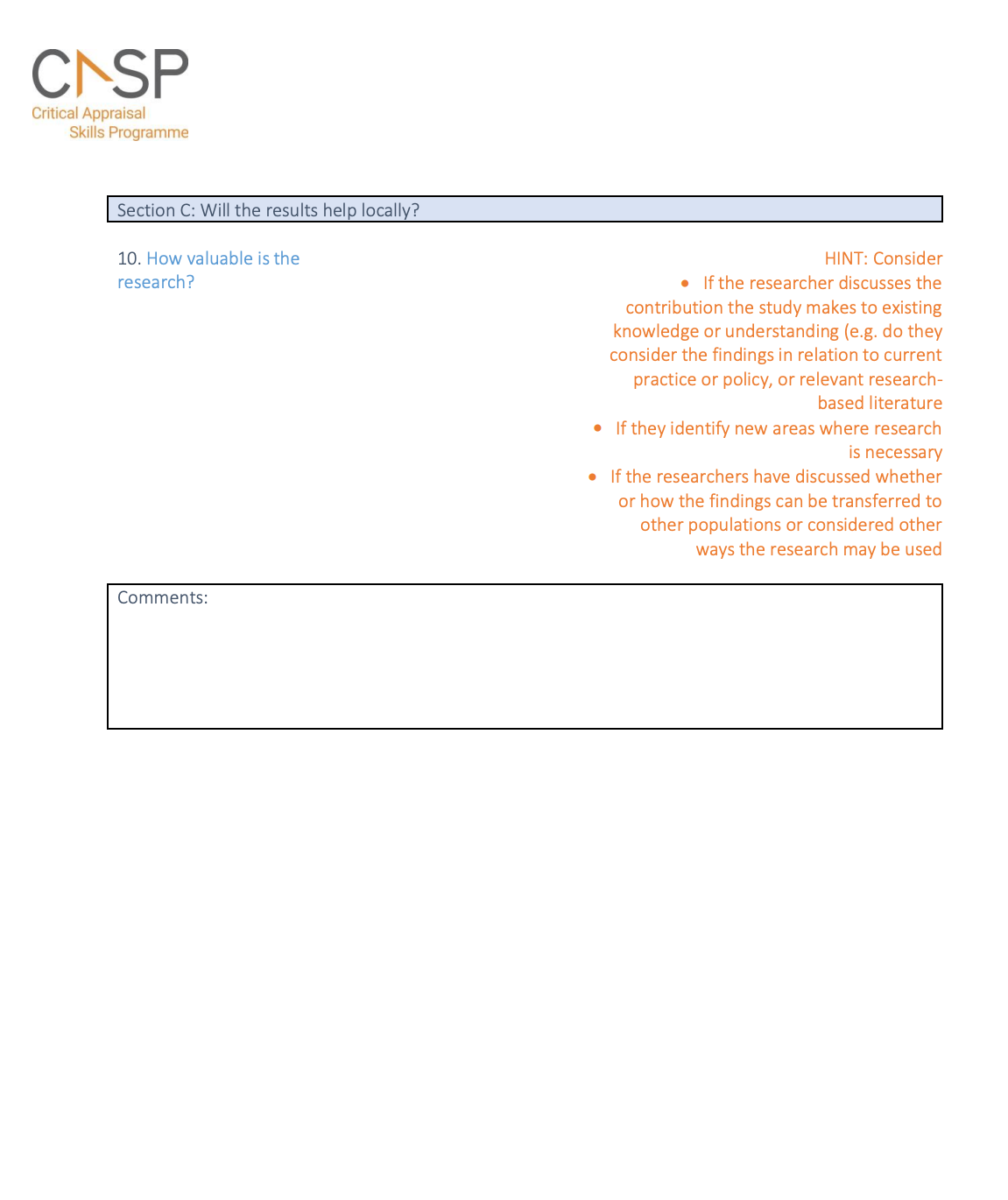


**Appendix F - Studies Requiring Discussion and Outcomes**

| **STUDY** | **CONCERNS AND DISCUSSION** | **DECISION** | **RATIONALE** |
| --- | --- | --- | --- |
| Fletcher-Randell (2022) | Analysed written materials; would add variety to the review, given varied type of data and location. Difficulty identifying and differentiating data from autistic mothers' first-hand accounts. | Excluded | Difficulty in identifying/differentiating data from autistic mothers' first-hand accounts. |
| Litchman et al., (2019) | Analysed written materials; would add variety to the review, given varied type of data and location. Difficulty identifying and differentiating data from autistic mothers' first-hand accounts. | Excluded | Difficulty in identifying/differentiating data from autistic mothers' first-hand accounts. |
| Rosqvist & Lovgren, (2013) | Analysed written materials; would add variety to the review, given varied type of data and location. Difficulty identifying and differentiating data from autistic mothers' first-hand accounts. | Excluded | Difficulty in identifying/differentiating data from autistic mothers' first-hand accounts. |
| Hampton et al., (2022a) | Included data from autistic and non-autistic mothers; findings and quotes from autistic mothers were clearly demarcated. | Included | Findings and illustrative quotes related to autistic mothers were clearly demarcated. |
| Hampton et al., (2022b) | Included data from autistic and non-autistic mothers; findings and quotes from autistic mothers were clearly demarcated. | Included | Findings and illustrative quotes related to autistic mothers were clearly demarcated. |
| Kwang-Hwang & Heslop, (2022) | Included data from autistic mothers and autistic fathers; majority were mothers, quotes specified participants' sex. | Included | Autistic mothers constituted the majority of participants, and illustrative quotes specified participants' sex, allowing for extraction and synthesis. |
| Marriott et al., (2022) | Included data from autistic mothers and autistic fathers; majority were mothers, quotes specified participants' sex. | Included | Autistic mothers constituted the majority of participants, and illustrative quotes specified participants' sex, allowing for extraction and synthesis. |
| Smit & Hopper (2023) | Included data from autistic mothers and autistic fathers; majority were mothers, quotes specified participants' sex. | Included | Autistic mothers constituted the majority of participants, and illustrative quotes specified participants' sex, allowing for extraction and synthesis. |
| Grant et al., (2023) | Mixed-method survey; qualitative findings were clearly distinguished from quantitative data, with illustrative quotes provided. | Included | Qualitative data were clearly distinguishable from quantitative data, with illustrative quotes provided. |
| Donovan (2020) | Based on the same study as Donovan et al., 2023; reported and discussed different findings. | Included | Consistent with PRISMA guidelines, similar records are considered unique and included individually (Page et al., 2021). |
| Donovan et al., (2023) | Based on the same study as Donovan et al., (2020); reported and discussed different findings. | Included | Consistent with PRISMA guidelines, similar records are considered unique and included individually (Page et al., 2021). |
| Gore et al., (2023) | Relevance of study exploring autistic working mothers' experiences. | Included | Relevant and specific to autistic motherhood and autistic mothers’ experiences and discussed other aspects of parenting, meeting the review’s eligibility criteria. |
| Pentz et al., (2023) | Relevance of study exploring autistic mothers’ experiences of a specific perinatal service. | Included | Relevant and specific to autistic motherhood and autistic mothers’ experiences and discussed other aspects of parenting, meeting the review’s eligibility criteria. |

**Appendix G - Detailed Study and Sample Characteristics**

| **NO** | **STUDY INFORMATION** | | **SAMPLE** | | **METHODS** | | | **FINDINGS** | |
| --- | --- | --- | --- | --- | --- | --- | --- | --- | --- |
|  | AUTHOR  YEAR  TITLE  COUNTRY  TYPE | PARENTING PERIOD  NATURE OF EXPERIENCE | POPULATION  SAMPLE SIZE  AND METHOD  RECRUITMENT  ELIGIBILITY | SAMPLE CHARACTERSTICS | DESIGN  EPISTOMOLOGY  RESEARCHER RELFEXIVITY | DATA  COLLECTION | DATA ANALYSIS | THEMES | SUMMARY |
| **1** | Benson (2023)  Perplexing Presentations Compulsory Neuro-normativity and Cognitive Marginalisation in Social Work Practice with Autistic Mothers of Autistic Children.  United Kingdom, Peer-Reviewed Article | No specific time period.  Social work services and interventions. | Autistic mothers (n=7). Purposive Sampling via social media and A-UK organisation. Inclusion: formal or self-diagnosis, subject to social work intervention 2014-2021 for reasons related to parenting. | Autistic mothers of autistic children with co-occurring differences (anxiety, phobias, eating disorders, encopresis, ADHD, hypermobility syndromes, learning disability) (n=7). Autistic mothers of non-autistic children (n=3). Analysed and reported data only from autistic mothers of autistic children (n=7). Indicated variation in age, social class, nationality although not formally reported aside from ethnicity: white (n=6), mixed-race (n=1) | Qualitative Design. Critical constructivist epistemology. Reflexive statement, locates self as an autistic mother to two disabled children, integrated own experiences of topic and value of being a “critical autism expert”. Meaningful participatory approach. Minimal reflexive strategies: triangulation, identifies limitation of position, no alternative perspective. | Semi-structured interviews (online). | Grounded Theory (Charmaz, 2014) and Situational Analysis (Clarke, 2005). | 1.Perplexing children and cognitive injustice, 2. Neurodivergent children and the battleground of school, 3. Support? Here is a parenting course. Not bloody boundaries again, 4. Epistemic authority and the ‘normal’ | Participants and their children are viewed as ‘perplexing’ through a neuronormative lens of social work scrutiny. Challenges and adverse experiences were located within participants rather than the wider systems, structures and processes which make autism disabling. |
| **2** | Burton (2016). Exploring the experiences of pregnancy, birth, and parenting of mothers with autism spectrum disorder.  United Kingdom, Unpublished Doctoral Thesis  Grey Literature | No specific time period.  Experiences of pregnancy, birth, and general parenting children birth-adolescence. | Autistic Mothers (n=7). Purposive Sampling. Recruited via organisations, charities, social media. | Age Range: 22-48 years. Child Age Range: 2-18 years. No. of Children: 1 (n=3), 2 (n=2), 3 (n=2). No. autistic children: 1 (n=2), 2 (n=1), 3 (n=10). Location: Midlands (n=6), Coastal Town (n=1), Ethnicity: White/British (n=7). Marital Status: Single (n=1), partner who lived elsewhere (n=1), in a couple (n=1), living with partner/husband (n=3), divorced (n=1). Child Custody: Two children adopted (n=2), Child temporarily fostered as a baby (n=1). Co-occurring Diagnosis (dissociative identity disorder, postnatal depression, bipolar disorder, obsessive compulsive disorder, anxiety, and paranoid personality disorder) (n=5). | Qualitative Design. Critical Realist. Reflects on critical realist stance, influence of power and interest in feminism. Meaningful participatory approach. Several reflexive strategies: field notes, credibility checks using peer-review group, transparency in theme development. | Semi-structured interviews (in person, online). | IPA (Smith, Flowers & Larkin, 2009) | 1.We are different 1.1. Discrimination and powerful others, 1.2. Internalised stigma, 1.3. Celebrating difference. 2 Negotiating difference 2.1. Power of diagnosis and understanding. 2.2. Fighting systems. 2.3 Universal parent experiences. 3. The role of the mother-child relationship. 3.1 Unique relationship with child.3.2 Child gives meaning and focus. 3.3. Relationship as agent of change, 4. Navigating the parenting journey, 4.1. The medical system, 4.2 Importance of trust and humanity in relationships with professional, 4.2 lack of control and uncertainty, 4.3 Significance of family. | Findings highlighted the connection autistic mothers have with their children and sensory and communication challenges. These challenges impacted their experiences throughout their parenting journey, and they perceived themselves differently to others. |
| **3** | Donovan (2020)  Childbirth Experiences of Women with Autism Spectrum Disorder in an Acute Care Setting.  United States, Peer-Reviewed Article | Pregnancy-Perinatal period (2-3 months).  Pregnancy, childbirth and delivery and care in acute care services and settings. | Autistic mothers (n=24). Purposeful Sampling. Recruited via social media. Inclusion: autistic women without complicated birth capacity to consent and participate in interview, vaginal /c-section delivery. in acute care setting. Exclusion: birth complications, baby removed from mother’s care, unable to consent. | Age Range: 29-65. Formal diagnosis or self-identified (n=24). Delivery age by decade: 20s (n=9), 30s (n=11), 40s (n=4). Race: White (n=20), multi-Racial (n=2), Unknown (n=2). Location: Australia (n=4), UK (n=3), US (n=17). Delivery Type: Vaginal (n=17), C-Section (n=7). Breastfed: Yes (n=19), No (n=5). Years since birth: 6m–4.99 (n=8), 5–9.99 (n=8), 10–14.99 (-n=1), 15-19.99 (n=3), >20 (n=4). Highest education level: Middle school (n=2), High school (n=4), Tech school (n=6), College (n=5), graduate (n=7). Marital status: Single (n=3), Married (n=16), Divorced (n=3), Separated (n=2). | Qualitative Interpretative Descriptive Design. Role of researcher and epistemological position unknown. Some stakeholder involvement. Several reflexive strategies to enhance rigour: field notes, audit trail, reflexive journal, member checks. | Semi-structured interviews (in person, online, telephone). | Qualitative Analysis Method (Knafl & Webster, 1988). | 1.Having Difficulty Communicating, 2. Feeling Stressed in an Uncertain Environment, 3. Being an Autistic Mother. | Participants childbirth experiences are more stressful than **non-autistic** mothers due to communication and sensory differences and pre-existing anxiety. Miscommunication with, and negative judgements nurses had negative implications (e.g., trouble conveying needs, alerting nurses to feeling unwell, increased anxiety, inadequate pain relief, frustration, feeling scared, almost unnecessary caesarean birth, decreased trust). This resulted in increased anxiety, preventing further attempts to communicate. |
| **4** | Donovan et al., (2023).  Yes, I can bond”. Reflections of autistic women’s mothering experiences in the early postpartum period.  United States, Peer-Reviewed Article | Pregnancy-Perinatal period (2-3 months).  Post-partum care and experiences in peri-natal period. | Autistic mothers (n=24). Purposeful Sampling. Recruited via social media. Inclusion: autistic women without complicated birth capacity to consent and participate in interview, vaginal /c-section delivery. in acute care setting. Exclusion: birth complications, baby removed from mother’s care, unable to consent | Age Range: 29-65. Formal diagnosis or self-identified (n=24).  Delivery age by decade: 20s (n=9), 30s (n=11), 40s (n=4). Race: white (n=20), multi-Racial (n=2), unknown (n=2). Location: Australia (n=4), UK (n=3), US (n=17). Delivery Type: Vaginal (n=17), C-Section (n=7). Breastfed: Yes (n=19), No (n=5). Years since birth: 6m–4.99 (n=8), 5–9.99y (n=8), 10–14.99 (n=1), 15 19.99 (n=3), >20 (n=4). Highest education level: Middle school (n=2), High school (n=4), Tech school (n=6), College (n=5), graduate (n=7). Marital status: Single (n=3), Married (n=16), Divorced (n=3), Separated (n=2). | Qualitative Interpretative Descriptive Design. Epistemological position unknown, no reflexive statement but researcher locates self in research context as experienced maternal–new-born nurse, and mother of three autistic adult children. Professional stakeholder involvement, not participant group. Several reflexive strategies used; clarified responses, data saturation, peer consultation throughout data analysis, field notes, audit trail, reflexive journal member checks. | Semi-structured interviews (in person, online, telephone). | Qualitative Analysis Method (Knafl & Webster, 1988). | 1.Having Difficulty Communicating, 2. Feeling Stressed in an Uncertain Environment, 3. Being an Autistic Mother, 3.1 breastfeeding, 3.2. mother-baby bonding experiences | Participants bonding experiences are similar to **non-autistic** mothers, they expressed love and concern for babies. Some needed more time to recover physically and emotionally before caring for babies. Stress of childbirth and caring demands were exhausting and overwhelming. Concerns within postpartum period and postpartum care included sensory differences, communication difficulties, trouble trusting nurses and negative judgements. |
| **5** | Dugdale et al., (2021). Intense connection and love: The experiences of autistic mothers.  United Kingdom, Peer-Reviewed Article | Childhood & Adolescent Period.  General Experiences of Motherhood. | Autistic mothers (n=9).  Purposive Sampling via research laboratory database, social media, local charities. Inclusion: 18+, formal diagnosis, self-identifying and/or awaiting assessment, parent to child. 5-15 years. Exclusion: Intellectual Disability (ID), Non-English Language. | Age Range: 27-44. Diagnosis: Formal (within 0-6 years of study) (n=8). Self-diagnosed/ awaiting assessment (n=1). Gender Identity: Female (n=9) Ethnicity: White/British (n=7), White/Welsh( n=1), mixed race (n=1). Marital status: Married (n=9) Education Level: NVQ Level 2 (n=3), BA/BSc (n=5), MSc (n=1). Employment: Self-employed (n=4), Employed (n=3), Full-time carer/parent (n=2). Co-Occurring neuro-developmental, mental health or Physical health diagnosis (n=6). Parenting: Co-parent with married partner, also birth/adoptive father (n=8), Separated from birth father, co-parenting with new partner and biological father of other children (n=1). Child Diagnosis: Formal Autism Diagnosis (n=7), Suspected autism (n=2). | Qualitative Design. No reflexive statement but researcher locates self in research context with professional experience. Meaningful participatory approach. Highlights philosophical underpinnings of IPA but does not explicitly state epistemological position. Several reflexive strategies: detailed, transparent coding, field notes, triangulation, reflexive journal. | Semi-structured interviews (online). | IPA (Smith, Flowers & Larkin, 2009) | 1. Autism fundamentally impacts parenting, 1.1.Shared diagnosis and similarities  Intrinsic part of life for better or worse, 1.2. Battle for the right support, 2.1. Misunderstood judged and dismissed, 2.2 Understanding autism within the family unit, 3. Development and acceptance, 3.1. Diagnosis, self-care, and self-acceptance, 3.2. Parenting as growth and adaptations, 4. The ups and downs of parenting, 4.1. intense connection, enjoyment, and rewards, 4.2 Managing children’s autism/other needs and impact on self. | Motherhood is a joyous experience. Autistic mothers have intense connections and closeness with children. Self-acceptance, self-care is important for personal growth. Autistic mothers’ challenges relate to autism, unlikely to be experienced by **non-autistic** mothers. Challenges include negotiating misunderstandings with others, managing demands and negative interactions with professionals. Negative interactions and lack of awareness, acceptance and support from professionals had a profound negative impact. |
| **6** | Gardener et al., (2016). Exploratory Study of Childbearing Experiences of Women with Asperger Syndrome  United States  Peer-Reviewed Article | Early motherhood; pregnancy, childbirth, and early postpartum period.  Experiences relating to this time period. | Autistic mothers (n=8). Recruited via distributing questionnaire to relevant stakeholders from Asperger’s Syndrome Community. Inclusion: self or formal diagnosis, relevant stakeholders from Asperger’s Syndrome Community. | Age Range: 27-52. Age of first baby:18-32 years (n=8). No of children: 1 (n=1), 1+ (n= 7). Marital Status During Pregnancy: Married (n=5), Couple (n=3) Diagnosis Status During Pregnancy: Unaware (n= 2), aware (n=5), unknown (n=1). Formal diagnosis pre-birth (n=2), Formal Diagnosis post-birth (n=4), Self-identified (n=2). | Qualitative Design. Questionnaire piloted to participants for feedback but instead analysed responses, justified this through detailed responses but no explicit discussion of ethical implications or rationale for not re-distributing questionnaire integrating feedback as intended. Role of researcher and epistemological position unknown. Minimal reflexive strategies: audit trail, compared results between researchers. Claims to support authenticity by deriving findings from data and using quotes, no evidence. | Qualitative questionnaire | Open and holistic coding and analysis methods (Saldana, 2009). | 1.Processing Sensations, 2. Needing to Have Control, 3. Walking in the Dark, 4. Motherhood on My Own Terms | Autistic mothers experience several challenges throughout pregnancy, birth and early motherhood including sensory challenges, lack of control, poor communication, difficulties adapting to motherhood and negative experiences with professionals. Professional interactions shaped positive or negative experiences. |
| **7** | Gore et al., (2023). ‘‘Maybe No One Knows We Need Help’’: Understanding the Experiences of Autistic Working Mothers in Australia.  Australia, Peer-Reviewed Article | No specific time period.  Experiences of employment and parenting and useful supports to manage everyday life. | Autistic working mothers (n=10). Recruited via social media.  Inclusion: female at birth, identify as women/non-binary person, Australian resident, speak and understand English, formally diagnosed with autism, parent, or caregiver of at least one child under 18 years old, currently/recently employed within last 12 months. | Age range: 34-50 (n=10). Diagnosed in adulthood, (n=9). Location: Inner Regional Australia (n=4), Major City (n=6). Age Ranges (mean = 42.7): 30-34 (n=1), 35-39 (n=1), 40-44 (n= 3), 45-49 (n=3), 50-54 (n=1). Gender: Female (n=9). Highest education level: BSc/BA (n=5), Diploma (n=2), MSc (n=2). Employment status: Permanent full-time (n=2), permanent part-time (n=5), self-employed full-time (n=1), Contract part time (n=1). Average weekly hours: 8 (n=2), 12 (n=1), 20 (n=1), 22.8 (n=1), 24 (n=2), 38 (n=1), 40 (n=1). Relationship status: Married (n=6), Single (n=2), Divorced (n=1), Repeated (n=1). Ethnicity/Race: Australian non-Indigenous ethnicity (n=8), English (n=1), Mixed race (Australian, Italian, Greek) (n=1). Co-occurring conditions anxiety (n=9), ADHD (n=5), depression (n=5). No. of Children: 2+ (under 18) (n=10) ,1+ neurodivergent children (n=9). | Inductive Qualitative Design  Subjective epistemology, relativist ontological perspective and provides reflexive statement. Locates self in research context and reflects on subjective influence as employed autistic woman, mother to three children (two neurodivergent). Participatory approach. Several reflexive strategies: reflexive diary and supervision, peer-review process in data analysis, member checking. | Semi-structured interviews. (online). | Inductive RTA (Braun & Clarke, 2021). | 1.‘Wellbeing: Work gives me purpose; discusses how employment supports mental well-being., 2. Challenges: It’s hard being an autistic working mother., 3. The invisible disability: Everyone thinks I look okay. | Participants have similar challenges to non-autistic working mothers including stress related to juggling multiple roles, however, have unique challenges related to gender and autism including poor understanding of maternal autism amongst employers and other professionals. Employment supports mental well-being. Negative judgements and inaccurate assumptions by professionals prevented them from seeking support. Challenges specific to being a working mother including caring for children and difficulties managing care and time when caring for neurodivergent children. |
| 8 | Grant et al., (2023). “It felt like I had an old-fashioned telephone ringing in my breasts”: An online survey of UK Autistic birthing parents' experiences of infant feeding.  United Kingdom  Peer-Reviewed Article | Early motherhood, maternity period.  Childbirth, infant feeding, and experiences of maternity services and associated health care. | Autistic mothers (n=141). | Autistic mothers responded to quantitative questions (n=152); autistic mothers responded to qualitative questions (n=141). Demographics based on (n=152). Age Range: 19-54. Autism Diagnosis Status: Diagnosed (n=88) Undergoing diagnosis (n=29), Self-Identified (n=35). Communication Preference: Speaking (n=128), Sign Language (n=1), Alternative and Augmentation Communication (n=1), Other (n=2). Autistic masking: Always/most of the time (n=103), half of the time (n= 29), Sometimes /never n=20) Gender identity: Cis (n=126), Non. binary (n=14), Other (n=10), Prefer not to say (n=2) Ethnicity: White (n=140) Mixed/multiple (n=5) Asian/Asian British (n=2), other (n=4) Prefer not to say (n=1) Disability: yes (n=116), no (n=33), prefer not to say (n=3). Disability impact: A lot (n=37), a little (n=84) Not (n=26) Prefer not to say (n=2). Support for disabilities: Strongly/somewhat agree (n=18), Neither agree/disagree (n=21), Strongly/somewhat disagree (n=111), Prefer not to say (n=2). Highest qualification: None (n=1), GCSE (n=12), A-levels (n=17), NVQ(n=21), BA/BSc (n=54), MSc (n=31) Doctorate (n=11), Other (n=5) Location: England (n=116) Scotland (n=11), Wales (n=20) Northern Ireland (n=5). | Mixed method design. Locates self in research context as two autistic researchers, involved two other autistic researchers in reviewing suitability of survey. No further reflection on potential influence, or bias. epistemological position unknown. Minimal reflexive strategies, 1+ analysts, illustrative quotes. | Online mixed method survey (open and closed questions). | Inductive TA (Braun & Clarke, 2021). | 1.breastfeeding, 1.1.motivation to breastfeed, 1.2. positive breastfeeding experiences, 1.3. breastfeeding challenges, 1.4 expressing, 1.5. stopping breastfeeding, 2. Formula feeding, 3. infant feeding support. | Autistic mothers have a strong desire to breastfeed. Mixed experiences were reported, challenges were ascribed to bodily changes and differences including sensory processing and interception. Receiving breastfeeding support was significantly associated with positive breastfeeding experiences, there were many negative aspects of infant feeding support, and this support did not appear to address Autistic bodily differences that impacted on breastfeeding. |
| **9** | Hampton et al., (2022a). A qualitative exploration of autistic mothers ‘experiences I: Pregnancy experiences  United Kingdom  Peer-Reviewed Article | Early motherhood; third trimester.  Pregnancy, physical and sensory experiences, and interactions with healthcare professionals. | Autistic mothers (n=24) and non-autistic (n=21) women. Recruited via existing research databases, social media, autism and pregnancy related support groups, magazines, and social media. Inclusion: over 18 years, self-reported formal diagnosis. | Demographics based on autistic women (n=24): Mean age: 31yrs 10 months. Range: 21-35 years. Formal diagnosis: (n=24) ethnicity: White (n=24), Education level: Undergraduate or above (n=14), A- level or below (n=10). Annual household income: >50000 (n=7), <50000 (n=17). Psychiatric conditions: No (n=8). Depression (n=2) depression and anxiety (n=7), OCD and anxiety (n=2), Other (n=5). Country: UK (n=19), USA (N=4), Ireland (n=1). No. of children (not including current pregnancy) 0, (n=18), 1(n=2), 2 (n=4) Pregnancy conditions: Gestational diabetes (n=5) Polyhydramnios (n=1). Mean AQ score 39.8 | Qualitative Design. Authors locate self in research context as two self-identifying autistic researchers and research teams roles, aimed to produce research to benefit the autistic community but did not reflection on specific influence or potential for bias during data collection or analysis. Epistemological position unknown. Some stakeholder involvement. Minimal reflexive strategies. | Semi-structured interviews (online, telephone, email). | Inductive TA (Braun & Clarke, 2006). | 1.physical and psychological impact of pregnancy, 1.1 The impact of sensory changes, 1.2 ‘The impact of other physical changes, 1.3. The emotional impact and social pressures of pregnancy, 2. the impact of formal and informal support, 2.1. Considerations around disclosure and professionals’ autism expertise, 2.2.Communication needs during antenatal care, 2.3 ‘Other met and unmet support needs , 2.4 The importance of informal support., 3. Fears and Hopes of Motherhood. 3.1.Birth-related fears and hopes’, 3.2. Anticipation of the challenges and benefits of parenthood. | Autistic mothers experienced heightened sensory and physical symptoms during pregnancy compared to non-autistic mothers. Autistic mothers were sometimes reluctant to disclose their diagnosis to healthcare professionals and felt that professionals lacked autism knowledge. While both groups appreciated clear information about their care, autistic participants further highlighted the need for detailed information and being given time to process verbal information. The autistic group also highlighted the need for sensory adjustments in healthcare settings. |
| **10** | Hampton et al., (2022b). Qualitative exploration of autistic mothers’ experiences II: Childbirth and postnatal experiences  United Kingdom  Peer-Reviewed Article | Early motherhood; pregnancy - 2-3 months post-birth.  Experiences of childbirth and parenting during post-natal period, post-natal health care and the benefits and challenges of parenthood. | Autistic mothers (n=21) and non-autistic mothers (n=25). Recruited via existing research databases, social media, autism and pregnancy related support groups, magazines, and social media. Inclusion (based on previous research study recruitment) included: pregnant people, autistic or non-autistic, with and/or without formal diagnosis.  Exclusion: no birth complication’s, twin pregnancy, no MRI contraindications, under 18. | Autistic mothers (n=21). Demographic based on (n=20)  Age Range: 21-35. Mean age of child in weeks: 10.76, Range: 8.29-13.57, Ethnicity: White (n=20). Education level: Undergraduate or above n=(11), A level or below (n=9). Annual household income: >50000 (n=6) <50000 (n=14). Psychiatric conditions: No (n=7), Depression (n=1), Depression anxiety (n=6), OCD and anxiety (n=2). Other (n=4). Country: UK (n=15), USA (N=4), Ireland (n=2). No. of children (not including current pregnancy): 0 (n=16), 1 (n=1), 2 (n=3) Type of delivery: Vaginal (n=9), Assisted (n=2), C-section (n=10). Mean gestational age at birth in weeks: 39 (n=18) Pregnancy conditions: Gestational diabetes (n=4), Polyhydramnios (n=1). Mean AQ score 40.60 | Qualitative Design. Role of researcher and epistemological position unknown. Some stakeholder involvement. Minimal reflexive strategies: 1+ analysts, member check by one autistic mother not included in study. | Semi-structured interviews (online, telephone, email). | Inductive TA (Braun & Clarke, 2006). | 1.Positive and negative birth experiences, 1.1  physical and emotional challenges of birth, 1.2  autism disclosure and accommodation around specific needs, 1.3 communication needs  other met and unmet support needs, 2. Rewards and challenges of motherhood, 2.1 motherhood as a rewarding experience, 2.2 hopes and expectations for child, 2.3 impact on day-to-day functioning, 2.4 other demands of motherhood, 3. Impact of formal and informal support, 3.1 professional’s autism expertise and accommodations around specific needs, 3.2 importance of building relationship with professional’s, 3.3 other met and unmet support needs, 3.4 importance of informal support. | Findings showed sensory aspects of childbirth could be challenging for autistic mothers. Autistic mothers stressed the importance of sensory adjustments and clear, direct communication from professionals during the birth. During childbirth and the postnatal period, autistic mothers sometimes felt that professionals lacked knowledge of autism, and this could hinder receiving appropriate adjustments. Several parenting strengths and challenges were identified. |
| **11** | Kwang-Hwang & Heslop (2022)  Autistic parents’ personal experiences of parenting and support: messages from an online focus group.  United Kingdom, Peer-Reviewed Article | No specific time period.  Experiences of parenting and parenting support. | Autistic mothers (n=5) and autistic fathers (n=2). Purposive sampling via National Autistic Society. Inclusion: parents diagnosed autistic; autistic child; sufficient written and spoken English without support; 18+, reliable internet access; capacity to consent. | Demographics for autistic mothers (n=5): Age Range: 36-53. Marital status: Separated/Divorced (n=2), Married (n=3) Ethnicity: White (n=5), Diagnosis: Formal (n=5). Age of Diagnosis: 30-56 years. Other Conditions: Learning Disability and Depression (n=1) Learning Disability (n=2), OCD (n=1) No. of children: 1 (n=3), 2 (n=1), 3 (n=1). No of autistic children aged 4-25 years: 1 (n=5) | Qualitative Design. Role of researcher and epistemological position unknown. Minimal stakeholder involvement (moderated focus group). Minimal reflexive strategies: 1+ analysts. | Focus group (online). | Inductive TA (Braun & Clarke, 2006). | 1.the ups and downs of parenting, 2. misunderstood and negatively judged, 3. battle for the right support. | Autism may not impact always on mothers parenting capacity, and, when it does, they can succeed in raising their children, especially their autistic children, if they are provided with appropriate support services. Their parenting style and capabilities were misunderstood by professionals who used traditional pathologising assumptions on parental capacity. |
| **12** | Lewis et al. (2021). Exploring the Birth Stories of Women on the Autism Spectrum  United States, Peer-Reviewed Article | Early motherhood; childbirth  Childbirth experiences | Autistic mothers (n=16) Convenience Sample. Recruited via online forums and social media. Inclusion: were eligible 18+, self-identifying as autistic, experienced childbirth. | Age range: 21-57 years. Self-identified (n=5), Formal diagnosis (n=11). One birth story (n=14), Multiple birth stories (n=2). Age Range at time of birth, 19-41 years, mean=27.1 years. Time since birth: range 6 months-26 years, mean 11 years. Gender: Women (n=16). Ethnicity: White (n=14), Location: US (n=6), UK (N=6), New Zealand (n=2). Aware of autism at birth (n=5). Aware of autism but did not disclose (n=2). Aware of autism post-birth (n=11). Participants who described their first birth experiences (n=14), vaginal birth (n=14). | Qualitative Narrative Design. Authors locate self by discussing role but no reflexive statement to discuss influence. Epistemological position unknown. Meaningful participatory approach: surveys individually tailored to participants. Several reflexive strategies: team of analysts, reflexive journals, peer reflection, independent and cross-check of data and narratives, member-checking. | Semi-structured interviews (in writing, online). | Narrative Analysis (Burke, 1945). | Tension most often occurred when actions taken by the health care team were out of balance with their approach to care which left autistic mothers feeling that their concerns were minimized, their wishes were ignored, and they were left out of critical communication and education. Participants also struggled when their own autistic traits such as sensory sensitivities, were out of balance with the birth environment which impaired their ability to communicate with providers and participate in the birth. | Poor communication, untreated pain, and sensory overload dominated the birth narratives of participants. Autistic mothers’ personal narratives suggested that the way they were treated by health care team members and the social and sensory stimuli in the birthing environment were most influential in shaping their birth stories. |
| **13** | Libster, (2023) Autistic motherhood: The experience of raising a non-autistic adolescent daughter.  University of California  United States, Peer-Reviewed Article | No specific time period.  Experiences of parenting non-autistic adolescent daughter, birth-adolescence. | Autistic mothers of adolescent daughters (n=7). Purposive Sampling. Recruited via poster advert to autism organisations and social media. Inclusion: formal or self-diagnosed autistic mother, daughter 10+ without autism diagnosis, verbally fluent in English. | Age range: 40-64. Time of self/formal diagnosis. age range 35-52. Education level: High school graduate (n=1), (Master’s/PhD) (n=6) Employment: full-time (n=3), part-time (n=2), self-employed (n=2). Location: US (n=4), Canada (n=1), England (n=2). Ethnicity: White (n=7) Relationship Status: Married (n=3), Divorced/Separated (n=3), Unmarried (n=1). Daughter age: 10-12 years (n=2), 13-18 years (n=3), 19+ (n=2). | Descriptive Pilot Study. Qualitative Design,  Participatory approach. Located self in research context as non-autistic researcher and non-mother, positionality statement, reflects on potential influence. Discusses epistemology of IPA clearly and theoretical basis of study. Meaningful participatory approach. Several reflexive strategies: reflexivity journal, 1+ analyst including lived experience experts. | Semi structured interviews (online). | IPA (Smith, Flowers & Larkin, 2009) | 1.Closeness in relationships, 1.1 expressed affection, 1.2. safety and support, 1.3 understanding mothers’ autism, 2. Parenting strengths, 2.1 problem solving skills, 2.2 positive strategies for managing conflict, 3. Identifying own social challenges, 3.1 understanding social dynamics, 3.2 friendships and social groups. 4.Building daughters' social skills. 4.1 concern about daughters’ social development. 4.2 opportunities for positive social interactions. | Autistic mothers have strengths and challenges and guide their children’s social development.  Strengths include affectionate loving relationships, understanding and supportive mother-daughter relationship, proactive approach to ensuring opportunities for daughters’ social development.  Challenges include understanding social dynamics, negative experiences with other parents, concerns about daughters' social development. |
| **14** | Marriott et al., (2022).  Parenting an Autistic Child: Experiences of Parents with Significant Autistic Traits.  United Kingdom, Peer-Reviewed Article | No specific time period.  Aim: to investigate the lived experiences of parents of autistic children who have significant autistic traits themselves | Autistic mothers (n=7), autistic fathers (n=1). Purposive Sampling. Recruited from NHS child mental health service and charity. Inclusion: scored over 32 on Autism Quotient, at least one child with formal, confirmed diagnosis of autism. | Demographics of autistic mothers: Age range: 26-50. Diagnosis: formal (n=2), undiagnosed (n=5). Co-morbid diagnosis: yes (n=2), no (n=5). | Qualitative Interpretative Descriptive Design, Participatory Approach. reflective account on role and potential impact, epistemological position unknown. Several reflexive strategies: Yardley’s quality criteria, reflective journals, supervision, parallel analysis, independent researchers to concur themes, audit trail and credibility checks. | Semi-structured interviews (online). | IPA (Smith, Flowers & Larkin, 2009) | 1.The interaction of parents’ and children’s autistic traits both helps and hinders parent–child relationships, 2. The personal impact of being a parent with autistic traits, 3. Home is a rare place of acceptance of autistic traits for parents and children, 4. Managing the complexities of professional services: struggling to be heard, believed, and supported. | Difficulties with parental mental health and navigating professional services. Novel participant experiences included the interaction between parental and child autistic traits helping and hindering their parenting; parents learning to manage their own autistic traits, and parents finding the home to be an accepting place of autism. |
| **15** | Morgan (2019). The autistic birth experience – Results from a survey. School of Medicine, Swansea University.  United Kingdom  Unpublished MSc Dissertation (Grey Literature) | Early motherhood; pregnancy- childbirth.  Experiences of pregnancy and childbirth | Autistic mothers (n=249). Inclusion: 18+, formal or self-diagnoses, pregnancy resulted in live birth. Recruited via social media advertisements and emails sent to authors contacts in relevant fields (midwifery and education). | Demographics collected, not formally reported: Year of birth, gender, sexual orientation, formal or self-diagnosis, ethnicity, nationality, country of birth, education level, employment, relationship status, manner of delivery, gestation age, postnatal child health complications, pre-pregnancy issues/co-morbidities. | Mixed method design. Contextualist stance within neurodiversity and social model theoretical context. Minimal self-reflexivity to discuss their role and influence. Meaningful participatory approach. Several reflexive strategies: detailed analysis, triangulation, member-checks, detailed analysis, and transparency of themes. | Online survey (open questions and quantitative rating scale). | Inductive TA (Braun & Clarke, 2006) | 1.Community and Isolation. 2.Privacy and Freedom  3.Diagnosis- Timing and Scepticism, 4. Retrospect- The Effect of Timing. 5. Exceptional Care | The adult autistic population of women who have given birth is evidently underdiagnosed and underserved by current medical care. Autistic women’s care experiences were largely negative and included poor-quality practices. Good quality practice examples included positive home transitions, consequences of poor practice included potentially unnecessary interventions and concerns about parenting ability. |
| **16** | Pentz, Cooke & Sharp., (2023). Experiences of women with autistic spectrum condition accessing the Brighton and Hove Specialist Perinatal Mental Health Service  United Kingdom, Peer-Reviewed Article | Peri-natal period.  Experiences of perinatal mental health care services. | Autistic mothers (n=5). Recruited via practitioners in service. Inclusion: patients on professionals’ caseload (pregnant or one-year post-partum with severe mental health difficulties), formal autism diagnosis or suspected and awaiting assessment. | Age Range: 24-33 years. Formal diagnosis (n=2) Awaiting assessment (n=3). | Qualitative Design. Researchers state role, no discussion of influence. Epistemological position unknown. Non-participatory approach. Minimal reflexive strategies: 1+ analysts. | Semi-structured interviews (online) | TA (Braun & Clarke, 2006). | 1.Interventions, 1.1. meeting format, 1.2 patient centred care, 2. Support of ASC characteristics, 2.1 clinician attitudes, 2.2 sensory support 2.3diagnosis and identification, 3. Practitioners’ Support, 3.1 patient-practitioner relationship, 3.2 availability. | Highlighted positive aspects of the service and barriers. Participants experiences were mixed. Positives include continuous support and flexibility around appointments. Negatives include online format of DBT group and delays in autism diagnostic assessments. |
| **17** | Radev et al., (2023). I’m not just being difficult . . . I'm finding it difficult': A qualitative approach to understanding experiences of autistic parents when interacting with statutory services regarding their autistic child.  United Kingdom  Peer-Reviewed Article | No specific time period.  Experiences With Statutory Services. | Autistic mothers of autistic children (n=10). Purposive Sampling via social media and autism database.  Inclusion: 18+, fluent in English, formal diagnosis of autism, parent of an autistic child currently attending mainstream school. Exclusion: not UK resident, autistic child was not in mainstream school; they had a co-occurring intellectual disability (ID). | Age Range: 35–40 (n=2), 40–45 (n=1), 45–50 (n=3), 50–55 (n=4). Time since diagnosis (years): Mean = 2.9 < 1(n=4), 1-2 (n= 2), 4-5 (n=3), >5 (n= 1). Ages of autistic child/children (years): 5–10 (n= 3), 11–17 (n=12). Ethnicity: White British (n=8), Mixed race (n=2). Education: NVQ (n=1), BSc/BA (n=4), MSc (n=1), PhD (n=3). Employment status: Student (n=1), Unemployed (n=3), Employed (n=6). Marital status: Married/Co-habiting with child’s father (n=8), co-parenting with child’s father (n=1), Single (n=1). | Qualitative Interpretative Descriptive Design Author locates self in research context, identifying autistic mother of autistic children, fails to discuss influence. Epistemological position unknown. Meaningful participatory approach. Several reflexive strategies: summarised and verified participants responses, analytical checklist, 1+ analysts, consulted with other researchers. | Semi-structured interviews (online and via telephone) | IPA (Smith, Flowers & Larkin, 2009) | 1.The wider system is the problem., 1.1feeling dismissed and unsupported, 1.2 The system is unjust, 1.3 need to fight for the right support, 2. Feeling judged and stigmatised, 2.1 use of language negative and misleading, 2.2 Training is not good enough, 2.3 Usefulness of disclosing diagnosis. | Overall “absolutely awful” experiences with systems. Systemic problems including unfair and discriminatory processes and professionals uninformed and outdated views about autism contributed to challenges including being dismissed, unsupported, and poor communication which led to self-reliance. Participants feared disclosing autism or were judged and treated negatively. |
| **18** | Rogers et al., (2017). Perinatal issues for women with high functioning autism spectrum disorder.  Australia, Peer-Reviewed Article | Pregnancy, Birth & Early Motherhood  Experiences of pregnancy, birth, maternity care, and post-natal period. | Autistic mother (n=1). Recruited via relevant autism organisations via online and newsletter adverts. | Age: 26, PhD Level Education, Diagnosed with Asperger’s aged 17. | Qualitative, case study design. Researcher reflexivity or epistemology not discussed; no reflexive strategies documented. | Semi-structured interview (online and via email) | TA (Braun & Clarke, 2006). | 1.communication and service difficulties 2. sensory stress, 3. Parenting challenges. | Findings suggest autistic women face challenges during pregnancy, birthing, and early mothering. These challenges evolve from perceptions of her from midwives and other caregivers. If a woman perceives that her midwife is judgemental about her, then she may withdraw from the care and support she and her baby need. |
| **19** | Sanchez (2023). Against the Against the norm' mothering: A reflexive thematic analysis of autistic motherhood  London South Bank University  United Kingdom, Doctor of Philosophy Thesis (Grey Literature) | No specific time period.  Experiences of being an autistic a mother, social expectations, and interactions with professionals and clinicians, | Autistic Mothers (n=12) Purposive Sampling. Recruitment via online advert and local charities.  ,  . | Age: 30s (n=3), 40s (n=6), 50s (n=2), 60s (n=1). Employed: (n=6), autistic children (n=12), Diagnosed pre motherhood (n=1) post motherhood (n=11). | Qualitative Design. Critical Realism. Researcher locates self in research and theoretical context and reflects on influence as autistic mother of an autistic child. Meaningful participatory approach. Several reflexive strategies: reflexive diary, reflective commentary, illustrative quotes and detailed, evidenced analytical process and illustrative quotes. | Semi-structured interviews (email, WhatsApp messenger) | RTA (Braun & Clarke, 2021). | 1.Identity: Knowing I’m autistic helps me to understand myself., 1.1. It was like a lightbulb! 1.2. Affirmation of my autistic identity has been helpful., 1.3. I didn’t recognise the red flags. 2.Masking: Masking is a real double-edged sword, 2.1. There’s a lot of pressure on mothers to ‘fit in’, 2.2. I’m the queen of camouflaging, 3. Support, Women like me ‘fall through the gaps’ of support, 3.1. It’s hard to get support from anyone who understands my kind of autism, 3.2. I’ve had to make my own support network for myself., 4. Mothering: A good mum wants the best for her children, 4.1. You have to squash down your own needs, 4.2. Being an autistic mother feels like having insider information, 4.3. ‘Against the norm’ mothering, 5. Motherhood: autistic mothers are judged and problematised by the same forces that police gender roles in society., 5.1. Mum is the one who keeps things in place, 5.2. We get blamed a lot for our kids, 6. Knowledge: -if you are autistic, it’s presumed that you don’t know anything about anything, 6.1. All sorts of myths float around to make up for outdated knowledge, 6.2. They just see us as mum, who knows nothing. 6.3. I have a need to know exactly what something is about. | Findings tell a story of the lightbulb moment of self-realisation as autistic, the challenges of masking and accessing support; the joys and difficulties of mothering alongside the expectations of motherhood and of finding ways to resist and kick back, creative solutions and developing expertise. It highlights that poor awareness, understanding and support of autistic mothers contributes to mother blame narratives despite autistic mothers being highly skilled at recognising and meeting their children’s needs. Findings also suggest autistic mothers are attempting to resist the neuro-normative narrative of ‘good mother’ and co-construct own ideal of ‘good autistic mother’. |
| **20** | Smit & Hopper, (2023). Love, Joy, and a Lens of Childhood Trauma: Exploring Factors That Impact the Mental Health and Well-Being of Autistic Parents via Iterative Phenomenological Analysis.  United Kingdom  Peer-Reviewed Article | No specific time period,  General Parenting Experiences, Influence of Childhood Trauma, Mental Health, and Wellbeing. | Autistic mothers (n=8), autistic fathers (n=1). Recruited via advert to social media, autism charities and forums, advert in university student research pool. Inclusion: parent/carer of 1+ child under 18; diagnosed (or self-diagnosed) as autistic, UK resident, self-reported good understanding of written/ spoken English. Exclusion: unable to give informed consent. | Age range: 34- 50 years. Demographic based on both groups: Sex: female (n=8), male (n=1). Ethnicity: White/British (n=8), Mixed Race (Irish and Asian) (n=1). Child autistic or awaiting autism assessment (n=9). Female formal diagnosis (n=4), Female self-identified (n=4), Male formal diagnosis (n=1). Diagnosis: Diagnosed post-parent (n=9). Co-occurring disability (n=1), Relationship status: Married (n=3), Co-habiting (n=3), Separated(n=1), Divorced (n=2). | Qualitative Design. Located self in research context, identified subjective influence and offered reflections of insider researcher status as autistic parent. Non-participatory approach. Acknowledged double-hermeneutic IPA approach but did not discuss epistemological position. Several reflexive strategies: reflexivity diary, illustrative quotes, detailed analysis. | Semi-structured interviews (online, WhatsApp messenger, included visual media, memes, cartoons, poems, blogs). | IPA (Smith, Flowers & Larkin, 2009) | 1.Identity and Purpose Love and Joy, 1.2 Personal Growth, 1.3 Close Connections, 2. Looking Through a Lens of Trauma, 2.1 Extreme Empathy, 2.2 Changing the Narrative, 2.3 Perfectionism, 3. External Factors, 3.1 Awareness and Acceptance, 3.2 Environmental Pressures. | Autistic parents had intimate parent-child connections. Children were sources of love and joy. Parents’ childhood trauma influenced parenting experiences; extreme empathy, perfectionism, drive to protect children from the same trauma. Professionals’ acceptance and awareness of autism was integral for positive outcomes during interactions. Mothers experienced pervasive sensory overload from their environments, related to losing coping mechanisms when they became parents. |
| **21** | Talcer et al., (2023). A Qualitative Exploration into the Sensory Experiences of Autistic Mothers  United Kingdom  Peer-Reviewed Article | Early Motherhood  Nature and impact of sensory experiences Pre-Natal, Childbirth and Post-Birth S | Autistic mothers (n=7). Volunteer Sample. Recruited via social media and Cambridge Autism Research Database (CARD)  Inclusion: female, formally diagnosed by professional living with child, UK resident, internet or telephone access, English language, 18+. Exclusion: known significant mental health problems, physical disability which limits mobility/ causes pain, brain injury/cognitive impairment. | Age Range: 30-55. Ethnicity: White (n=6), Unknown (n=1), Diagnostician: Psychiatrist (n=4), Psychiatric Nurse (n=1). Neurodevelopmental: OT (n=1), Doctor (n=1). Co-morbidities: N/A (N=3), Yes (Anxiety, OCD, Depression, Dyslexia, dyspraxia, IBS, Migraines) (n=4). No. of children: 2 (n=5), 1 (n=1), 3 (n=1). No of autistic children: 1 (n= 4), 2 (n=1), 0 (n=2). | Qualitative Design Authors locate self in research context as inside researchers, Occupational Therapist and Sensory Integration Practitioner, discussed advantages and drawbacks. Epistemological position unknown. Meaningful participatory approach. Several reflexive strategies: 1+ analyst, member checking, reflective journal. | Semi structured interviews (telephone). | Thematic Analysis (Braun & Clarke, 2006). | 1. Antenatal experiences, 1.1: pregnancy, 1.2: severe sickness, 1.3 labour & birth 2. Sensory experiences in motherhood, 2.1 auditory, 2.2. tactile, 2.3body awareness, 3. The impact of sensory processing difficulties, 3.1 social, 3.2. work, 3.3.mental health/anxiety, 3.4 overwhelm/fatigue., 4. Strategies and needs, 4. used strategies.  Needs, 4.2 Diagnosis., 4. 3 late diagnosis, 4.4 not being diagnosed, 4.5 benefits of diagnosis. | Autistic mothers with sensory processing difficulties have extreme and pervasive sensory challenges which exacerbated levels of stress and anxiety making many aspects of motherhood challenging. It impacted multiple facets of their mothering roles, including social and work roles, mental health, fatigue levels, ability to plan and organise day-to-day routines.  Helpful strategies include lessening effects of their sensory processing difficulties including downtime, asking for support, and linking with other autistic mothers. |
| **22** | Wilson & Andrassy (2022). Breastfeeding Experiences of Autistic Women  United States, Peer-Reviewed Article | Early motherhood, breastfeeding period.  Breastfeeding experiences. | Autistic mothers (n=23). Voluntary Purposive Sampling. Recruited via social media support groups. Inclusion: 18+, did not require legal guardian, formal/self-diagnosis, breastfed at least one infant. Exclusion: Non-English-speaking, unable to consent. | Ethnicity: Caucasian (n = 20). Marital Status Married (n=17), Children 2+ (n=18). Education college degree (n=9), master’s (n= 7). Bachelor’s (n=4). Location: US (n=14), UK (n=7), Canada (n=1), New Zealand (n=1). | Qualitative Phenomenology Design. Researcher role and epistemological position unknown. Several reflexive strategies: audit trail, detailed analysis, 1+ analyst and de-brief group, member checks. | Semi-structured interviews (online, telephone, text, email). | Thematic Analysis (Braun & Clarke, 2006). | 1.Intense Sensory Perception, 1.1.Overstimulated, 1.2.Over-touched, 1.3 Overwhelmed, 2.Focused Determination, 3. One Size Doesn’t Fit All. | Autistic adults can have social interaction and expressive communication differences. Nurses can promote positive communication and provide appropriate care through supportive action. |
| **23** | Winnard et al., (2022). Motherhood: Female Perspectives and Experiences of Being a Parent with ASC  United Kingdom  Peer-Reviewed Article | No Specific Time Period (pregnancy, birth-adolescence)  Experiences of ‘being a parent’. | Autistic mothers (n=4) and autistic non-mothers (n=4). Purposive Snowball Sampling. Recruited via advert to autism support group and social media. Inclusion: 18+, female, formal diagnosis of autism, English language. | Demographics based on both groups: Age Range: 28-63 (mean 41.5). Diagnosis age range: 9-50 years old (mean 28.9). Location England (n=7), Scotland (n=1). Children: Autistic (n=3) non-autistic (n=1). Partner for childcare support (n=8). | Qualitative Design. Researchers located self in research context, as assistant and qualified psychologists, positionality statement. Epistemological position unknown. Several reflexive strategies: reviewed positions, reflexive supervision, reflexive journal, 1+ analysts, documented theme development, quality standard tool. | Semi structured interviews (online, telephone, in person). | IPA (Smith, Flowers & Larkin, 2009) | 1.Parenthood: Fun & Games, 2. Support: Giving & receiving, 3. Routine & Structure, 4. Sensory Sensitivities, 5. Interaction, 6.Unique Insight. | There are benefits and challenges of being an autistic parent. Autistic specific skills and traits are associated with strength, resilience, love, nurture, routine, and sensory differences. Positives include parent-child bond, unique ability to relate to autistic children, being experts by experience to support child to learn, and develop. Challenges include battling for help and support from statutory services, struggling with changes to routine, sensory needs, and multiple demands. |

**Appendix H - Detailed Quality Appraisal**

**Peer Reviewed Studies**

***High Quality (9-10)***

Eleven studies were deemed “high” quality. Two were assigned 10 points (Gore et al., 2023; Libster, 2023), six 9.5 points (Dugdale et al., 2021; Lewis et al., 2021; Marriott et al., 2022; Radev et al., 2023; Smit & Hopper, 2023; Talcer et al., 2023) and three 9 points (Benson 2023; Grant et al, 2023; Winnard et al., 2022). These studies demonstrated transparency and coherence between their aims, design, and methods and the researcher’s indicated position. Authors located themselves in the research context by providing reflexivity statements and/or critically reflected on their potential influence throughout various aspects of data collection, analysis, interpretation and reporting of findings. However, only Benson (2023), Gore et al., (2023) and Libster (2023) explicitly stated the epistemology underpinning their study. Others alluded to this by discussing the philosophical underpinnings of their approaches. Almost all studies involved those with lived experience in various aspects of the research. These factors increase the trustworthiness of findings and the extent to which they privilege, reflect, and represent participants views. All but one (Winnard et al., 2022) discussed pertinent ethical considerations specific to the nature of the topic and population and detailed steps to address potential issues, accounting for its lower quality rating.

Of the ten papers, two specifically explored autistic mothers experiences during early motherhood (Grant et al., 2023; Lewis et al., 2021), one had a mixed focus (Winnard et al., 2022) whilst the remaining eight generally explored experiences beyond early motherhood (Benson, 2023; Dugdale et al., 2021; Gore et al., 2023; Lewis et al., 2021; Libster, 2023; Marriott et al., 2022; Radev et al., 2023; Smit & Hopper, 2023), suggesting increased trustworthiness of studies which have explored autistic mothers’ experiences beyond early motherhood.

***Moderate Quality (7.5-8.5)***

Eight studies were considered “moderate quality”. Five were assigned 8.5 points (Donovan 2020; et al., 2023; Hampton et al.,2022; 2022b; Wilson & Andrassy, 2022), one 8 points (Kwang-Hwang & Heslop, 2022) and two 7.5 points (Pentz et al., 2023; Rogers et al., 2021). All these studies failed to sufficiently report their epistemological position or adequately discuss researcher reflexivity including use of self-reflexive strategies, thus minimising how the researchers influence may have impacted various aspects of the study design, data collection, analysis, interpretation and reporting of findings. There was also inadequate detail provided of analytical processes, with most providing a more generic overview of the steps involved and a brief summary of their specific process. These limitations had obvious implications for the trustworthiness of the findings and raise slight concerns about the extent to which participants voices were privileged and findings represent their views (Thomas & Harden, 2008). Moreover, whilst ethical approval was clearly stated for all studies, several failed to provide sufficient detail or discuss potential issues of exploring a sensitive and potentially distressing topic.

All studies except for Kwang-Hwang & Heslop, (2022) explored autistic mothers’ experiences in early motherhood, these moderately rated papers increase trustworthiness of extant literature exploring autistic mothers earlier parenting experiences, indicating the synthesis should not be solely weighted towards evidence of later motherhood experiences.

***Very Low Quality (6-)***

Only Gardener, (2016) study was deemed “very low quality”, scoring 5.5 points. This study had several concerns, largely relating to the unintended, unplanned nature of the study which had multiple implications on its quality across most domains. **These included issues** pertaining to recruitment, data collection and analysis, interpretation and reporting of results, ethical considerations, and absence of researcher reflexivity or epistemological stance. The methodological limitations of this study pose risk to its rigour and reduce the trustworthiness of findings, suggesting less weight should be placed on this study within the synthesis. This study focused on autistic mothers’ experiences of early motherhood. Given the majority of papers shared this specific focus and were predominantly of moderate quality, and findings were consistent with all other studies, it was not considered to significantly impact the overall synthesis.

**Grey Literature Studies**

***High Quality (9-10)***

All three non-peer reviewed studies were deemed “high quality”. Two were assigned 10 points (Burton, 2016; Sanchez, 2023) and one assigned 9.5 points (Morgan, 2019). All papers demonstrated transparency and coherence between the aims, design, and methods and authors gave clear rationales for their choices. Burton (2016) and Sanchez (2023) clearly located themselves in the research context. They provided reflexive positionality statements, critically discussing the epistemology or theoretical basis of their studies and reflected on their potential influence on, and throughout the study. Morgan (2019) highlighted their positionality but failed to adequately reflect on this. All studies used reflexive strategies used to counter any bias throughout various aspects of the study and detailed analytical processes, reporting themes and summarising findings clearly and in context of the studies aims and the relevant context. Ethical considerations were highly detailed and autistic people’s involvement was prioritised.

Overall, the grey literature papers quality ratings suggest they were of high methodological quality. Findings were also consistent with those reported in peer-reviewed literature, increasing confidence in the findings of these papers. Furthermore, all authors were associated with reputable educational establishments which had approved submission of the papers in partial fulfilment for awards of higher education and/or professional accreditation **such as** MSc, PhD, DClinPsy. These studies adhered to the central tenets of scientific rigour and had undergone various academic and ethical peer-review processes and were suitable for both this review and the CASP checklists.

**Appendix I – Additional Illustrative Quotes**

| **DESCRIPTIVE THEMES** | **ILLUSTRATIVE QUOTES** |
| --- | --- |
| **ANALYTICAL THEME 1: THE EMBODIED AUTISTIC EXPERIENCE OF MOTHERHOOD** | |
| **“SUPER PARENT POWERS”** | - *“Their difference gave them strengths ... they could understand their needs in a way that parent without ASD might not be able to… “it is a strength in a lot of ways it’s like a super parent power”* (Burton, 2016) - *“I went literally everywhere to get the information I needed and craved …. now I have an almost encyclopaedic understanding”.* (Grant et al., 2023) - *“I’ve persevered with the breastfeeding … because of my autism I made it into a bit of a special interest and was reading everything and researching everything”* (Hampton et al., 2023). - *“Some described their determination as a breastfeeding obsession… I was obsessed-had a one-track mind, I kept with it because that’s how my brain is wired…I have to research, but once I do I go full force”* (Wilson & Andrassy, 2022). - *“They described their parent–child relationships as secure, and supportive (Hwang-Kwang & Heslop, 2023).* - *“All participants spoke about understanding their child and their child’s needs, which was often described as an “instinct” (Tanya) or “intuneness” (Sarah), suggesting a sense of effortlessness”* (Marriott et al, 2022). - *“Parents with ASC were reported to be able to “positively and helpfully” impress routine and … due to their own innate strive for structure”* (Winnard et al., 2022). - *“Participants highly valued their “insider information” (Katharine), which they felt enabled them to better understand what their children were experiencing and where they might need extra support”* (Sanchez et al., 2023). - *“Participants expressed incredible feelings of love for their children. Their children were sources of joy and unconditional love”* (Smit & Hopper, 2022) - *“Seeing his progression and celebrating the wins and the small things that other parents would take for granted for us is a huge celebration”* (Dugdale et al., 2021). - *“They looked forward to motherhood with excitement, ‘I really, really can’t express how excited I am about being able to meet her and cuddle her’”* (Hampton, 2022a) - *“Becoming a parent had awakened positive emotions”* (Smit & Hopper, 2023). - *“Spending time’ and ‘being’ with their child(ren) was deemed as enjoyable and fun aspects of parenting”* (Winnard et al., 2022). - *“The majority of mothers described how their relationships with their daughters were characterized by safety and support”* (Libster, 2023)   *“To prepare for motherhood, many women in this study had consulted books and viewed videos during the prenatal period”* (Donovan et al., 2023)  *“Being autistic enabled them to use knowledge, describing themselves as ‘focused’ (Tara); using ‘determination and my autistic features’ (Kayleigh); and being a ‘typical autistic person [who] did lots of research’ (Kelly). One participant commented ‘forewarned is forearmed, so to speak isn’t it.. . it’s knowledge that breaks down all the barriers for everything’ (Chloe) … they perceived themselves to be at an advantage being autistic”.* (Radev et al., 2023)  *“Most parents found or created their own support and resources”* (Marriott et al., 2022).  *“Several participants reflected on their analytical thinking, in-depth ‘research’ (Ava, Alice), and planning of ‘practical matters’ (Lucy) as positive and linking with their ‘autism trait[‘s]’ (Ava). For example, participants linked such behaviour to a ‘special interest’ (Alice), ‘a desire for certainty’ (Zoe), or to a ‘black and white thinking’ (Lucy) style resulting in a desire to be ‘all in’ (Lucy) or to be ‘the best parent I [they] could’”* (Dugdale et al., 2021) |
| **MOTHERHOOD AMPLIFIES**  **“THE**  **AUTISTICKY BITS”** | - *“Being flexible to, and managing the multiple and often changing, unpredictable parenting tasks alongside other demands was referred to as a specific challenge for this population of parents”* (Winnard et al., 2022) - *“Participants discussed the relentless nature of parenting having a negative impact on their own well-being”* (Marriott et al., 2022) - *“Routine was hard to achieve as there were ‘demands being put on you all the time’ (Ava). Similarly, others struggled with the constant nature of demands”* (Dugdale et al., 2021). - *“They commented on the relentless nature of motherhood, such that they had little time to themselves”* (Hampton et al., 2022a) - *“Most women commented about difficulties processing the sensations associated with pregnancy and about other enhanced sensitivities to touch, light, sounds, and interaction”* (Gardener, 2016). - *“Another mother attributed her difficulty with social interactions to the cognitive demands that were required, explaining that “there's just so much multitasking, auditory processing, trying to figure out the rhythm and the timing of the conversation”* (Libster, 2023) - *“Autism-related challenges of breastfeeding were frequently reported … primarily centred around the bodily differences that Autistic people have including interoception, proprioception and sensory processing”.* (Grant et al., 2023). - *“Participants reported feeling ‘‘exhausted,’’ ‘‘tired,’’ ‘‘overwhelmed,’’ and/or decreased executive functioning because they are juggling employment, parenting, household tasks, and self-care…” I’ve been exhausted for years, but it’s gotten to a whole new level…’I get burnt out from trying to do it all”’* (Gore et al., 2023). - *“The sense of overwhelm that resulted from trying to manage multiple competing demands often resulted in burnout and participating mothers often sacrificed their own plans as a way to reduce the load”* (Sanchez, 2023). - *“All participants reported that pregnancy made their sensory experiences noticeably more heightened, and for some, this never really decreased to baseline following the birth of their child”* (Talcer et al., 2023) - *“Our participants spoke about taking on their children’s emotions as if they were their own” …. most participants felt the persistent need to be perfect: ‘‘The perfectionist trait is there strong and true. There’s the constant worry and overthinking of: Am I doing it right? or Am I doing my best for him?”* (Smit & Hopper 2023). - *“Difficulties related to... feeling they did not fit in ‘the normal mum’s club’ (Sophie). … Many described struggling with the impact on themselves from parenting, such as lack of sleep and exhaustion”* (Dugdale et al., 2021) - *“The autistic group additionally found it challenging to adjust to rapid changes in body size and shape”.* (Hampton, 2022a) - *“The women often put their children’s needs first, sometimes, to the detriment of their own: “it’s stressful erm so yeah, I mean I always have to squash my needs down.... you do you get a kind of burn out and you can cope with much less”* (Burton, 2016) |
| **SIMILAR YET DIFFERENT** | - *“All participants reflected on how being autistic was a part of them and how their strengths and weaknesses in relation to parenting were, therefore, inherently related to being autistic. Consequently, many highlighted the impossible task of ‘teas[ing] out the autisticky bits from the non-autisticky bits’ (Leah). All participants reflected that being autistic resulted in both strengths and weaknesses for parenting”. (Dugdale et al., 2021).* - *“Mothers were able to recognize their difficulties in understanding social dynamics and socializing with other parents, who often ignored and excluded them”.* (Libster, 2023) - *“I just constantly felt like I was failing as a parent. Because you couldn’t get everything right all of the time. That’s just not the case with being a parent. Um, which. And I think that’s taken me a long time to realise that. I’m sure neurotypical parents probably realise that six weeks in, but it’s taken me nearly 16 years to realise!”* (Smit & Hopper, 2023). - *“By the nature of their sensory or social experiences with others, these women felt different to the majority and had to adapt to find a way of surviving in a world that they felt did not appreciate diversity. Having children increased this contact with the mainstream world, which may have increased this sense of difference when comparing themselves to other parents… Though putting children’s needs before parent’s needs is a universal parental experience it may occur more consistently for mothers with ASD due to extra challenges around managing sensory difficulties and communication”*. *(*Burton, 2016) - *“From a sensory point of view, breastfeeding was also reported by some to be a positive experience”.* (Grant et al., 2023) - *“Some reported that their sickness felt “different” when they compared their experiences to other neurotypical mothers”.* (Talcer et al., 2023). - *“Because I rely on my intellect… I can't show up in a way that other people would perceive as being present. Doesn't mean I'm not there. It just doesn't show up in a way that other people would think I was there”* (Libster, 2023). - *“Two women … found breastfeeding helped them feel calmer than usual…” And to be honest, I felt happy when I was (breastfeeding). And less anxious, yeah, the world just could go away*” (Donovan et al., 2023). - *“I just couldn't keep up with my peers and the social expectations of new parents. I hated trying to make new friends and felt incredibly isolated because I was different”* (Morgan, 2019). - *“The sensory challenges were greater or otherwise different”* (Gardener, 2016). - *“Participants perceived the outside world as rejecting of being different, for both themselves as being “unconventional” parents (Rosie), …They discussed difficulties with the “pressure to be, like, a certain kind of mother” (Rosie) and to “run with the pack” (Sarah). Sarah described having “almost like two worlds […] There are things that…that are just parts of me that I don’t…share widely”, suggesting she felt unable to act naturally outside her home”.* (Marriott et al., 2022) |
| **ANALYTICAL THEME 2: NAVIGATING THE NON-AUTISTIC** **WORLD AS AN “OTHER” MOTHER** | |
| **“M-OTHERED”; SYSTEMIC, STRUCTURAL, AND SOCIETAL OTHERING** | - *“I’ve been asked by a couple of the midwives how I think I can be a mum if I’m autistic. … I would never put my daughter in danger, but there’s been very much a feeling that that would be a possibility” (*Hampton et al., 2022b) - *“The traumatizing part comes from the doctor or nurse rolling their eyes or saying settle down or you’re interfering or making me feel bad about the reaction I just had, which I probably couldn’t control anyway. Um, that’s when you are made to feel stupid or bad because you didn’t just comply automatically”.* (Donovan, 2020) - *“No matter how many times I tried to explain that what they were doing was aggravating the Asperger's Syndrome because the hospital was stressing me out, they would not listen. According to my husband, I was doing extremely well with the pregnancy until that hospital got their hands on me and then I deteriorated rapidly…I've noticed that the more I strongly asserted my decisions on how to raise my own child, the more they put my behaviour down to some dysfunctional behaviour due to Asperger's Syndrome. In other words, they were questioning my function as an effective parent. …They seem to be a bunch of bullies”.* (Rogers et al., 2017) - *“Rebekah described feeling overwhelmed by the sensory environment, which led to her becoming nonverbal, which then led to her feeling left out of communication, which made her feel even more overwhelmed”* (Lewis et al., 2021) - *“They* [social workers] *said this clearly to staff at school, ‘they are not trying hard enough”.* (Benson et al., 2023) - *“Perplexing presentations were consistently identified as problematic by social workers with explanations and solutions being proposed in accordance with neuro-normative knowledge and standards*” (Benson, 2023). - *“All participants spoke of feeling misunderstood, judged, or dismissed, leading to difficulties in receiving support for themselves or their child. Participants reflected on how being misunderstood was typically linked to their autistic traits”.* (Dugdale et al., 2021) - *“What’s written on the paperwork is ‘we're very concerned that he thinks he's autistic’. As if it's the worst thing in the world to see yourself as autistic. So, my perception of social workers and the word ‘autism’ is that they think that it's some really bad thing to have, and they've got a really negative opinion on what autism is. And I think that that's a negative thing for our community”* (Benson, 2023). - *“I felt the professionals involved at my 2nd child’s birth threatened to take me to hospital against my will …I was forced to undergo a parenting assessment before birth”* (Morgan, 2023). - *“Participants talked about feeling judged and stigmatised through the language used indicating outdated and stereotyped understandings resulting from poor autism training. “There is a worry as an autistic parent, because there is a lot of stigma there, if you say that you’re struggling, that can be a lot more detrimental than it can be for other parents (Kim)… This feeling of being unsupported had a direct impact on their experience of parenting causing them to feel like a ‘rubbish mum because I feel like we didn’t have enough fun’ (Kim) … with time being spent on navigating systems to get the right support” (*Radev, 2023). - *“Becoming a parent brought the women into contact with powerful others who, due to their professional status, had the power to judge their parenting rights and capacity but also exposed them to society’s judgements about parenting. …. Anne unexpectedly had her parental rights removed …, “I thought they were the people who knew best … I thought well I’ve done nothing wrong you’ve got no evidence so why take her? …lack of resources and opportunities to demonstrate parental capacity resulted in Anne and Melissa having their children removed and they felt powerless to challenge this …. The mothers had to demonstrate that they needed no help at all, or their parental capacity would be questioned …. This led to some of the mothers feeling unable to ask for help leaving them in an even more powerless position”* (Burton, 2016). - *“Felt like they were constantly being rejected from mental health, educational and social services.… This was often due to families’ difficulties not being deemed severe enough. Parents described services instructing them to “come to us when it’s serious” (Kate) which invalidated their distress. Tanya experienced the lack of help as feeling like they were “marooned at sea much of the time”* (Marriott et al., 2022). - *“Some no longer sought assistance …due to the lack of support and understanding from health professionals … ‘‘And I was telling them again, and again, I can’t cope. And I just, I was just dismissed. So, I didn’t seek support because after that, I just, I just learned to cope with it’’* (Gore et al., 2023) - *“Participants were often not judged as ‘good’ parents due to labelling on autism…A person from [a charity organisation] said, you couldn’t do, or couldn’t be a parent because of your autism” (*Kwang-Hwang & Heslop, 2022) - *“Acceptance and awareness of autism was a factor that was out of our participants’ control. Schools and health care professionals held the power to either positively or negatively impact our participants’ mental health through acceptance and awareness (or lack thereof)”* (Smit & Hopper, 2023). |
| **RARE POSITIVE EXPERIENCES** | - *“It depended on the individual clinician. … I was lucky with my maternity care as I had access to a service where I had a regular midwife and had my scans in health centres rather than the stress of the hospital”* (Morgan, 2023). - *“One participant recounted how important the support of one teacher in the school playground had been to her: It wasn’t judgemental)”* (Radev et al., 2023) - *“Participant 4 mentioned her practitioner prewarning her of plans being changed helped to prevent distress” (*Pentz et al., 2023) - *“Some participants shared that members of their health care teams were helpful in adjusting the environment to meet their sensory needs in ways that enhanced their birth experiences and outcomes, thus maintaining balance”.* (Lewis et al., 2021) - *“Georgia remembered the nurses being very “accommodating” with her desire to start breastfeeding as early as possible. Mary … recalled one … The nurse assisting her was caring and had a gentle approach that was comforting”* (Donovan, 2023) - *“Some felt that lack of awareness could be compensated for by an individualised approach: “[My midwife] doesn’t have a lot of experience of autism but she listens to what I have to say about my experiences and then she adapts”* (Hampton 2022a). - *“When they moved me around, they put something over my eyes so I wouldn’t be blinded. They told me exactly when people were going to come and who was going to come. They tried to give me my own midwife where possible, so I saw the same person all the time and they told me when they were changing. They gave me my own room, so I didn’t have to go on the ward. I can’t fault them”.* (Hampton 2022b). - *“Five women spoke about the importance of having family support and working as a team with their partners or carers”* (Gardener, 2016) - *“Five of the women reported good experiences with professional staff that were characterised by empathy, being listened to, time taken to understand the mothers’ needs and a focus on the relationship. When this relationship worked it could have a significant impact on the woman: “it made a massive difference a massive difference it had an impact on the way I saw things, the way I saw her”* (Burton, 2016) - *“She highlighted the strengths of one medical staff member who recognised her unique character. During my whole time in the hospital, I found most of the healthcare people woefully inadequate with the exception of two people”* (Rogers et al., 2017) - *“Being autistic or parenting autistic children or additional needs often improved understanding in social support networks, leading participants to feel as though they had an ‘ally’ (Grace) or supportive other”.* (Dugdale et al., 2021) - *“Many participants relied on their partners: “if [my husband] is around obviously that helps because we sort of do the ‘divide and conquer”* (Marriott et al., 2022). - *“Most participants reported making links with other autistic mothers online was of great benefit as they could be open about how they were feeling, not feel judged and develop a positive identity as an autistic mother”* (Talcer et al., 2023). |
| **MAKING SENSE, SURVIVING & EMPOWERING A NEW NORM** | - *“Participants often reflected how their feelings or needs and outward expression of these were ‘two very different things’ (Emily), leading to multiple misunderstandings. This mismatch led to difficulties with professionals as they assumed participants were ‘aggressive’ (Grace) or experiencing ‘anxiety’ (Oliva, Zoe) when they were ‘upset’ (Grace) or had a need for ‘certainty’ (Alice). This often resulted in dismissals or not being ‘taken seriously’ (Olivia)”* (Dugdale et al., 2021). - *“I saw a psychologist for depression (…) as that what everyone thought it was. I was also surprised by the psychologist I had not picking up on autism; they really need training on it”* (Morgan, 2023). - *“They kept referencing Anne Heggerty in the jungle and saying, ‘I know a bit about autistic women now, because Anne Heggerty in the jungle said this and now, I understand what you’re saying’. These are health professionals and they’re getting their information from ‘I’m a Celebrity Get Me Out of Here’, otherwise they’d have had no understanding of me at all. I just thought that was the most awful thing, that reality TV is educating people who have the power to possibly take my child away from me”* (Hampton 2022b) - *“In spite of concerns about judgement and awareness of how mothers are expected to mother, participants typically mothered their children in ways that met their needs, even if this went ‘against the norm’. Participants spoke of being “less conventional” (Lydia), “unfettered by social norms” (Rosie) and doing “what I thought was right” (Emily) in their mothering practices, demonstrating both a rejection of standard mothering practice and confidence in their own mothering”* (Radev et al., 2023). - *“It isn’t just that we autistic people struggle to fully understand non-autistic people, but that non- autistic people also struggle to understand us and, as a result, might mis-read our intentions and, indeed, our discomfort or distress”* (Sanchez, 2023) - *“Many women feel misunderstood. Professionals look at breastfeeding in a very neuro-typically based way. We are all different. Some of us are very dramatic, others are highly logical and literal. Remember one size doesn’t fit all …. I think we need understanding, acceptance, respect, and nurturing towards us. Don’t view us as broken and less, but different. It’s a neurological difference-don’t judge us if we seem less attached emotionally”* (Wilson & Andrassy, 2022). - *“Participants reframed challenging aspects of autism to carry properly out parenting. They strongly believed they can look after their children, especially autistic children, if they receive appropriate support. Importantly, participants emphasised that this does not mean that all autistic parents should be offered additional parenting support to carry out their parenting “Not every autistic is the same or require same treatment or help”* (Kwang-Hwang & Heslop, 2022) - *“Mary and Melissa managed the lack of control and lack of explanation by resisting the interventions from staff. For Mary this meant crossing her legs during labour in protest as a result of the staff trying to give her pethidine when she did not want it, which delayed the birth process”* (Burton, 2016). - *“Participant 2 thought that the first time her neurodivergence was discussed, it was not given enough attention as she felt her practitioner did not have adequate knowledge on the subject”* (Pentz et al., 2023) - *“Many of the parents wanted to provide information and hope for Autistic adults who are just beginning parenthood and cannot find the resources and information they need online. Other parents indicated that their motivation to share their experiences of ableist discrimination in accessing child/parent services was to educate professionals about Autistic parents’ needs and prevent others from experiencing the harm they and their children had sustained”.* (Smit & Hopper, 2023) - *“People really need to listen to Autistic parents . . . we’re not thick, we see things from a different perspective”.* (Dugdale et al., 2021) |
| **ANALYTICAL THEME 3: RECALIBRATING IDENTITIES** | |
| **MIXED FEELINGS ABOUT BECOMING A MOTHER** | - *“Parenting their children led to self-growth and acceptance … Many reflected that although change or flexibility was something they could struggle with (as linked to their autism) parenting helped them become more accepting of this …. For some, adapting themselves to meet their children’s needs was a positive experience resulting in them feeling ‘proud of [themselves]”* (Dugdale et al., 2021). - *“Motherhood brought a new sense of identity and confidence that the women had not experienced before) … children were seen as motivators for change, giving the mothers a reason to put themselves in situations that they might not usually enter and to try new things”* (Burton, 2016). - *“Participants experienced a shift in identity when they became parents. They acknowledged the loss of the person that came before and described a sense of personal growth. Before having children, Judith experienced low self-esteem and self-recrimination. That changed when she saw herself through her children’s eyes: …Most participants felt the persistent need to be perfect”.* (Smit & Hopper, 2023). - *“Participants spoke about the positive aspects of parenting. Hilary illustrated: “I am very glad I became a parent. Because it has changed me as a person. Most of it for the better…”. Some highlighted learning skills which may not have come naturally to them, such as learning to “chit chat” (Sarah) or becoming less rigid. For example, Hilary described having to be "more laissez-faire with things...my control aspects"* (Marriott et al., 2022) - *“Participants overwhelmingly were positive about being a mother, sometimes to their own surprise, and confident in their mothering abilities. However, this was tempered to an extent by awareness of the expectations of mothers and the fear of being judged against standards they could either not meet or choose to disregard because they considered them irrelevant* *or harmful”* (Sanchez et al., 2023). - *“Childbirth often represented an unknown …worries around uncertainty were often linked to a desire for predictability, ‘there’s the uncertainty of when it’s going to be and how long it’s going to take and what’s going to happen, that uncertainty is adding to my fear of it’* (Hampton et al., 2022b) |
| **UNDERSTANDING AND INTEGRATING AUTISM** | - *“Participants typically had their children before realising they were autistic … participants wished they had known they were autistic or had been diagnosed earlier in life, often evoking a sense of grief, “I feel a lot of grief over my diagnosis, I think it’s related to difficulties I had throughout my life that could be explained or understood differently through an autistic lens” (Lydia). Alongside re-appraisal, some participants also expressed regret at past choices and missed opportunities, bemoaning the lack of support which might have improved their quality of life”*. (Sanchez, 2023). - “*They insisted that their diagnosis provided valuable parenting skills and understanding, especially with their autistic child”* (Kwang-Hwang & Heslop, 2022) - *“Others experienced anger and injustice for their own childhood, and not getting the additional support they had needed as a child with additional needs”* (Marriott et al., 2022). - *“All participants received a diagnosis of autism after already becoming a parent”.* (Dugdale et al. 2021) - *“All participants reported getting their Autism diagnosis after becoming a mother … diagnosis was a powerful and important step in understanding why they found certain situations difficult, led to greater self-acceptance and allowed them to develop new coping strategies… Most participants reported feeling weird or different before getting their diagnosis, leading to the assimilation of a negative self-concept with many reporting a negative impact on their mental health because of being undiagnosed”* (Talcer et al., 2023). - *“Because I didn’t realise, I was autistic until I had children, I’ve done a lot of self-blaming. So, like it’s probably a positive actually”* (Smit & Hopper, 2023) - *“It was like a lightbulb… When talking about their lightbulb moment, participants commonly described a backdrop of having never really understood themselves, and of now having greater insight and self-awareness …Clare described a similar re-framing, commenting that diagnosis “probably saved my life” (*Sanchez, 2023). - *“Polly thought that her experiences of depression during her last pregnancy and difficulties bonding with her baby were in part due to not having the diagnosis and this understanding of herself”* (Burton, 2016) - *“Another major challenge raised by participants related to autism as a ‘‘hidden disability’’ and they reflected on how this impacted their ability to access support”.* (Gore et al., 2023). |
| **SHARING AUTISTIC IDENTITIES** | - *“Participants talked about feeling judged and stigmatised through the language used indicating outdated and stereotyped understandings resulting from poor autism training. There were mixed views among participants on whether it was beneficial or detrimental to disclose their own diagnosis*…*Some who had disclosed experienced a reaction indicating professionals had changed their view … somehow viewing them as less capable because of the diagnosis” (*Radev, 2023). - *“We as mum's still having to fear outing our diagnosis because they'll bring in social services if we mention it. Autists can possibly be adequate parents, can they? …I've been very well aware that one should not share your diagnosis with any medical staff if possible as it's more often not well received, or the response is way more harmful than helpful”* (Morgan, 2023). - *“The same participant experienced high levels of stress in a hospital’s A&E department due to a lack of awareness and acceptance of autism: ‘‘I couldn’t do anything about it because they were talking to me like they were CBeebies presenters…they kept talking about me being ‘‘someone with autism… As a result of this kind of treatment, participants expressed a fear of disclosing their diagnosis”* (Smit & Hopper, 2023). - *“Participants had mixed views on the usefulness of disclosing their own diagnosis due to the mixed responses from professionals with some feeling it was an important part of spreading awareness and being listened to while others were fearful of being judged as people would ‘use it against me’ (Tara”* (Sanchez, 2023). - *“Sometimes diagnosis led to a reversal from this ‘disregarded’ position in relation to understanding their child’s support needs, to being taken ‘seriously’ (Emily); linked to professionals viewing this as legitimate expertise”* …D*isclosing their diagnosis sometimes created further misinterpretations or negative judgements, …, such as professionals negatively judging participants ability to ‘cope’ (Ava) or parent and viewing them as ‘cold’ (Lucy)…. for many, this stigma led to an internalised belief of not being a ‘good enough parent’ (Olivia). ... for some this felt like being constantly ‘pushed away’ (Sophie) by professionals and for others it was experienced as not being believed accused of ‘making stuff up”.* (Dugdale et al., 2021) - *“Having an ‘invisible disability’’ dissuaded some participants from disclosing their autism at work and from requesting accommodations to cope with challenges: ‘‘you almost feel safer if I say I’ve got a headache or a migraine versus this is something that impacts me on a daily, you know, day to day and part of my life’’.* (Gore et al., 2023). - *“Participants who disclosed their diagnosis to professionals did so to bring about improvements in care, while those who did not worried that professionals would react negatively, ‘some medical professionals think that Asperger’s is a kind of hypochondriac fake excuse disorder so I’m afraid that if I brought up other concerns maybe they would treat me differently” (*Burton 2016) - *“Participants sometimes felt that disclosure was met with disbelief due to professionals lacking knowledge of autism among women, ‘I had a doctor the other day say, ‘I’ve worked with autistic kids, and you’re not like them’. And I was like, ‘OK, I’m probably not, and probably they’re mainly boys as well”* (Hampton 2022a) - *“Some participants felt professionals were dismissive of their autism diagnosis, and this was sometimes linked to a lack of autism awareness, ‘I don’t think people have a knowledge of it really, it’s just a word that they think they know what it means, I don’t really think they know how to put that into practice’ (Irene, autistic)” (*Hampton 2022b) - *“Identifying as an autistic person led to medical professionals making assumptions about my parenting skills and led to interventions which were inappropriate, and which increased my stress levels and caused me emotional distress before, during and after the birth”* (Morgan, 2019) |
